# Tracking collective emotions in 16 countries during COVID-19: a novel methodology for identifying major emotional events using Twitter

**DOI:** 10.3389/fpsyg.2023.1105875

**Published:** 2024-03-25

**Authors:** Apurv Chauhan, Vivek Belhekar, Surbhi Sehgal, Himanshu Singh, Jay Prakash

**Affiliations:** ^1^Institute of Psychiatry, Psychology & Neuroscience (IoPPN), King's College London, London, United Kingdom; ^2^School of Humanities and Social Science, University of Brighton, Brighton and Hove, United Kingdom; ^3^Department of Applied Psychology, University of Mumbai, Mumbai, India; ^4^School of Business and Law, University of Brighton, Brighton and Hove, United Kingdom; ^5^Department of Computer Science and Engineering, Texas A&M University, College Station, TX, United States; ^6^Silence Laboratories, Singapore, Singapore

**Keywords:** collective emotions, emotions during COVID-19, Twitter, time series, NLP, anxiety in COVID-19

## Abstract

Using messages posted on Twitter, this study develops a new approach to estimating collective emotions (CEs) within countries. It applies time series methodology to develop and demonstrate a novel application of CEs to identify emotional events that are significant at the societal level. The study analyzes over 200 million words from over 10 million Twitter messages posted in 16 countries during the first 120 days of the COVID-19 pandemic. Daily levels of collective anxiety and positive emotions were estimated using Linguistic Inquiry and Word Count's (LIWC) psychologically validated lexicon. The time series estimates of the two collective emotions were analyzed for structural breaks, which mark a significant change in a series due to an external shock. External shocks to collective emotions come from events that are of shared emotional relevance, and this study develops a new approach to identifying them. In the COVID-19 Twitter posts used in the study, analysis of structural breaks showed that in all 16 countries, a reduction in collective anxiety and an increase in positive emotions followed the WHO's declaration of COVID-19 as a global pandemic. Announcements of economic support packages and social restrictions also had similar impacts in some countries. This indicates that the reduction of uncertainty around the evolving COVID-19 situation had a positive emotional impact on people in all the countries in the study. The study contributes to the field of CEs and applied research in collective psychological phenomena.

## Introduction

Psychology has examined emotions primarily as an individual-level phenomenon. Even when considering group-based emotions, an individual's emotional experiences are regarded as an outcome of their psychological belonging or social membership to the given group (Mackie et al., 2000; Mackie and Smith, 2018). Before the individualization of social psychology (Farr, 1980), there had been considerable interest in collective-level psychological phenomena, including emotions, their transference, and their impact on individuals. For instance, Le Bon (1895, p. na) noted that crowds develop ideas and sentiments, and a “collective mind is formed, doubtless transitory, but presenting very clearly defined characteristics”. Similarly, Durkheim (1915) discussed emotional effervescence and collective emotional excitement in the totemic ceremonies of the Uluru and Kingilli people. More recently, there has been a renewed interest in how group memberships shape individual experiences of emotions due to social identification or bonds between members of the group (Kim, 2016). Despite their name, these emotions remain a type of individual emotion - the individual is still the unit of assessment as the appraisal of the event still remains tied to the individual (Goldenberg et al., 2020).

On the other hand, in situations such as wars or national tragedies, an examination of macrolevel affective processes beyond the smaller group-based emotions becomes necessary (Goldenberg et al., 2020). This has led to the development of the notion of collective emotions (CEs), which do not associate the experience of emotions with group memberships. Instead, they consider that people belonging to a collective may experience an emotion for a range of reasons, not limited to shared identities or bonds. CEs arise in a society in response to events such as wars, national tragedies and triumphs, or indeed other events that dilute the boundaries of identification between groups (Bar-Tal et al., 2007; Goldenberg et al., 2014). Research has shown that CEs tend to cut across identities to engulf entire collectives. For instance, Garcia and Rimé (2019) examined Twitter posts of over 60,000 users in the aftermath of the terrorist attacks in Paris in 2015 and found a long-term increase in lexical indicators for solidarity and pro-sociality. CEs emerge not only in real time but also when collectives recall a significant event. For instance, in Kim's (2016) study several years after 9/11, CEs developed amongst participants discussing the trauma of the terrorist attacks.

**Figure 1:**
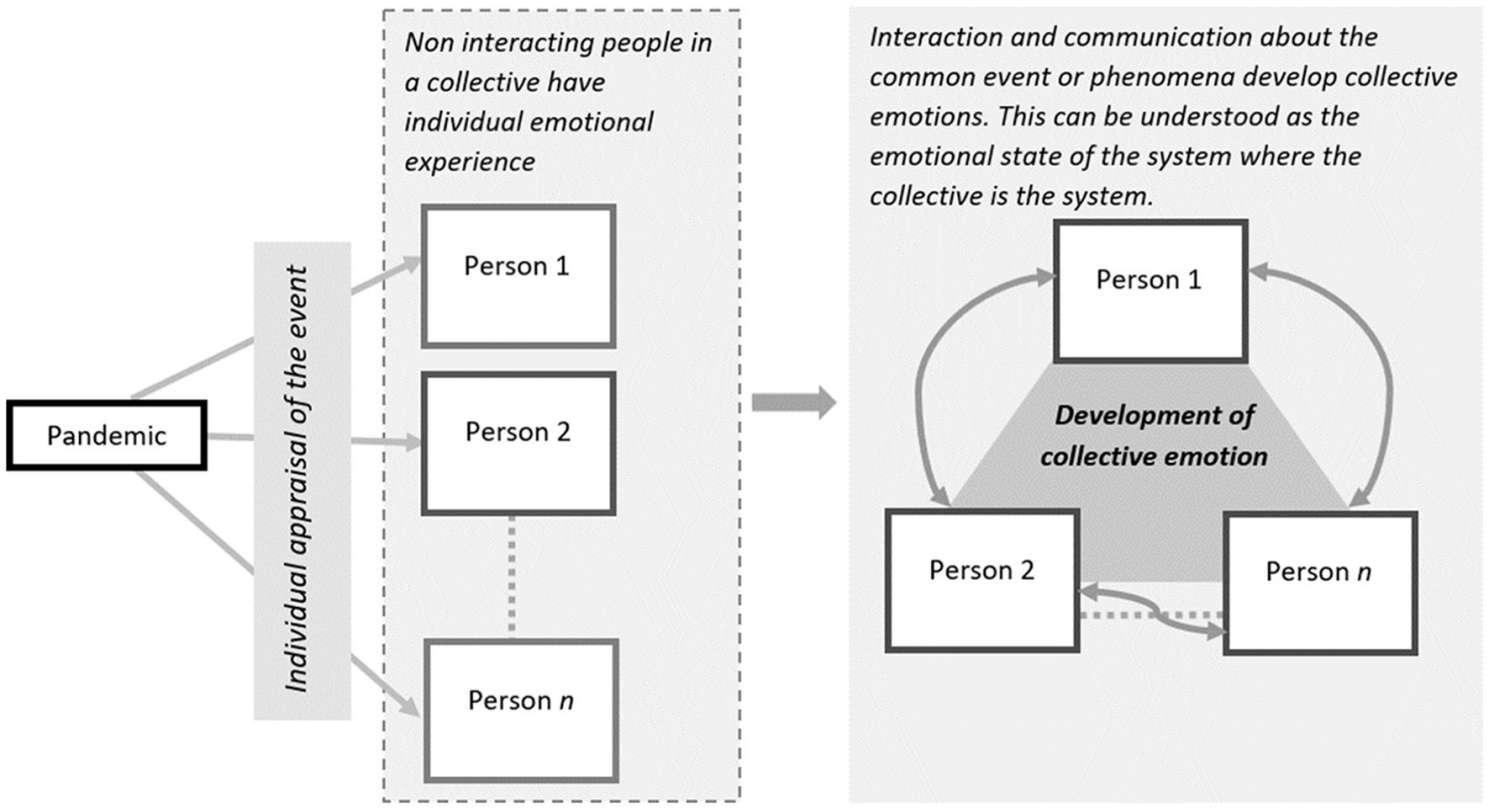
Development of CEs during COVID-19 pandemic.

In other words, CEs emerge in broader group contexts and, unlike individual level emotions, reflect the emotional state of a collective responding to the same event, phenomenon, or situation. CEs capture the overall emotional states of the systems where individuals activate, amplify, or reflect each other's emotional experiences, resulting in a predominant emotional state. Let us take a hypothetical event that has the possibility of evoking emotional experience in a collective: India winning the Football World Cup. If the collective at hand is of *n* individuals who watched the final separately on their TVs and remained in a communicative black hole, each of them would individually appraise the event and have an emotional experience. However, in a world where people interact, the emotional experience of the event will also be driven by *the experiences of others*. In a cricket-mad country, Person A may not care about what the country achieves in football. Yet, by virtue of being embedded in the social world where others do, they may eventually experience joy, pride, or other similar emotions. In summary, when a collective system develops a certain emotional state, individuals activate each other, and instead of individual appraisals, the collective state becomes the relevant trigger for emotional experiences. Figure 1 demonstrates these points. What is more, the Internet and modern technology, more than ever before in human history, have enabled near-real-time conversations about events in the social world. This makes the emerging field of CEs extremely pertinent to what Sheldon Stryker referred to as *sociological social psychology*. This study makes two significant contributions in this field. First, it makes a contribution by outlining an approach to measuring CEs from what people post on the Internet, and developing daily estimates of CEs in large collectives. Its second and more important contribution is of developing a novel application of CEs to explore the embeddedness of individuals in the collective and the social. The research develops a new approach to identifying events in the social world that have significant impacts on the emotional states of collectives.

## Methods

Three main methodological issues needed to be tackled in the project. First, an event sufficiently powerful to lead to the development of collective emotions needed to be identified. The COVID-19 pandemic adequately fits this criterion. The spread of the disease had an unprecedented impact on life globally and was marked by almost daily changes to public advisories in most countries. Lockdowns and restrictions on civil liberties affected the work and financial circumstances of people across the world (Bell and Blanchflower, 2020; Brewer and Gardiner, 2020; Nicola et al., 2020) and altered social norms and everyday lives (Alon et al., 2020; Dryhurst et al., 2020; Haleem et al., 2020; Nivette et al., 2020; Tagat and Kapoor, 2020; van der Westhuizen et al., 2020). More than any other new infectious diseases of the recent past, the rapid spread of COVID-19 was a cause of tremendous anxiety, alarm, and uncertainty (Goodwin et al., 2011; Mayor et al., 2013; Idoiaga Mondragon et al., 2017) around the world, and a number of studies have noted the pandemic to be an extremely emotional event (Andrade, 2020; Galea et al., 2020; Groarke et al., 2020). Given the high levels of public uncertainty and near daily changes in government advisories early in the pandemic, the study looked at CEs during the first 120 days^1^ of the COVID-19 pandemic.

Second, a source of data for estimating collective emotions in response to the said event needed to be identified as CEs develop out of interactions and communications between people dealing with a phenomenon of collective significance. Most debates, discussions, and conversations about the pandemic took place on the Internet as social restrictions and the nature of the disease prevented people meeting others in real life. One of these online public spaces was provided by Twitter, which is widely used for exchanging news, opinions, and feelings in real time. An online space such as Twitter, which people can access at all times, provides a natural means for the contagion, amplification, and reactivation processes and properties of CEs (Goldenberg et al., 2014, 2020). To illustrate, a person expresses their anxiety about the pandemic at time *t*. Their anxiety decays naturally, like all individual-level emotions, but on reengaging with Twitter, where the CE of anxiety remains high, they may experience a reactivation of their own anxiety. These reasons made Twitter the ideal landscape for examining the development of CEs, and not surprisingly, it has been used in other research on CEs previously (e.g., Garcia and Rimé, 2019).

Third, the project uses countries as the level of the collective at which CEs were tracked. The spread of the disease, hospitalizations, infections, and mortality rates were most commonly discussed at the level of countries. Similarly, the response to the pandemic—lockdowns, restrictions on civil liberties, support for workers and families—also took shape at the level of countries. For these reasons, this research considered countries to be the most appropriate level of the collective for studying CEs in response to the pandemic.

The following sections describe the methodological steps and analytic procedures.

### Extraction of country-level textual data from Twitter

A large publicly available dataset of tweets about COVID-19, maintained by Georgia State University's Panacea Lab, was used (Banda et al., 2020). In line with the privacy regulations, this dataset only provided an identification code (tweet ID) for individual messages posted on Twitter (henceforth, tweets). The dataset did not include retweets. Hydrator^2^ was used to process tweet IDs and extract text message content along with other associated metadata. At the time of conducting this procedure (Oct–Dec, 2020), nine percent of tweets in the dataset had either been deleted or made publicly unavailable by people who posted them. From each individual tweet ID, the associated text message, the country from which it was posted, and the date of its posting were extracted. For each date, words from individual tweets were combined to form aggregate corpora of words used on a particular date in each country—the study refers to them as *metatext*. To illustrate, 10,178 unique Twitter messages from the UK were extracted for 15 March 2020 with a total of 254,203 words. These 254,203 words formed the *metatext* for the UK for this date.

The project began with 25 countries with high numbers of Twitter users. Considering the use of natural language processing through the Linguistic Inquiry and the Word Count program to be undertaken for the next stage (described later), word-volume-based criteria were established with two rules for the inclusion of data from a country. *First*, in order to systematically eliminate countries with poor data quality, only countries with a minimum average of 5,000 words per day (over the 120-day period) were included. Austria, Costa Rica, Ethiopia, Switzerland, and Tanzania did not meet this criterion and were dropped from the study. *Second*, early in the pandemic, the number of tweets related to COVID-19 were low. This would result in some countries meeting the first criterion but having poor data at the start of the series. To maintain consistency between data from different countries, the latest start date of an uninterrupted series was set at 1 March 2020. Uninterrupted series were defined as at least seven consecutive calendar dates of 5,000 words in a country's daily tweet corpus. Brazil, Chile, and Peru were dropped because their uninterrupted time series started after 1 March 2020. The 16 countries that were finally included in the study covered five WHO-designated regions: Argentina, Canada, Colombia, Mexico, the United States of America (USA), and Venezuela from the Americas; France, Germany, Spain, and the United Kingdom (UK) from the European region; Nigeria and South Africa (SA) from the African region; India and Singapore from the East Asian region; Australia and New Zealand^3^ (NZ) from the Eastern Pacific region. Supplementary material 1 provides the details of the data preparation procedures and the software used. The original dataset had a systematic error on 9 February 2020, 28 February 2020, and 29 February 2020. These dates were treated as missing values and were imputed using the AMELIA package (Honaker et al., 2011) in R. Supplementary material 2 provides details of the imputation process.

The final textual dataset used for estimating CEs contained over 200 million words from more than 10 million Tweets posted from 16 countries. See Table 1 for details.

**Table 1:** Total number of tweets from each country from the start of time series.

**Country**	**Uninterrupted series start date**	**Length of uninterrupted series**	**Total words in the dataset (in millions)**	**Mean daily size of tweet corpus (in 1,000 words)**	**Language of the lexicon used**	**Lexicon hit rate (see text footnote ^2^)**
Argentina	27/01/2020	113 days	7.99	70.72	Spanish	75.59
Australia	27/01/2020	113 days	4.99	44.12	English	71.23
Canada	27/01/2020	113 days	6.89	60.96	English	69.58
Colombia	22/02/2020	85 days	2.84	32.66	Spanish	74.74
France	27/01/2020	113 days	6.12	54.18	French	55.44
Germany	01/03/2020	79 days	2.00	25.31	German	69.27
India	27/01/2020	113 days	29.93	264.86	English	63.55
Mexico	27/01/2020	113 days	5.76	50.93	Spanish	72.87
New Zealand	02/03/2020	78 days	1.49	19.08	English	71.96
Nigeria	27/01/2020	113 days	11.49	101.67	English	64.12
Singapore	28/01/2020	112 days	1.55	13.87	English	66.22
South Africa	01/03/2020	79 days	2.89	36.53	English	71.99
Spain	23/02/2020	86 days	3.72	43.30	Spanish	74.69
UK	27/01/2020	113 days	24.61	217.82	English	71.8
USA	27/01/2020	113 days	95.22	842.65	English	71
Venezuela	27/01/2020	113 days	4.42	39.14	Spanish	72.02
**Grand total** **=** **211.91**				**119.86**		

### Estimating collective emotions

The analytic stage of the research began by developing daily estimates of collective anxiety and collective positive emotions in each country during the 120-day period. The choice of estimating anxiety was driven by the well-documented anxiety-provoking impact of the pandemic globally (Pashazadeh Kan et al., 2021). The umbrella category of positive emotions was used as a foil for comparing levels of anxiety. Daily levels of both emotions were estimated using the Linguistic Inquiry and Word Count (LIWC) application (Tausczik and Pennebaker, 2010). LIWC is the benchmark tool in text analytics with a well-established validity in measuring emotional expression, and its rating of the emotional content of the text has been demonstrated to correspond to human ratings (Alpers et al., 2005; Kahn et al., 2007; Hall et al., 2020). LIWC's psychologically validated dictionary was used to search for lexical indicators of anxiety and positive emotions (Windsor et al., 2019). Each date in each country was searched with LIWC separately and a daily time series estimate of collective anxiety and collective positive emotions was developed. Repeating this process on the text corpora from each country separately provided us with daily time series estimate of the two CEs in all sixteen countries.^4^ Table 1 provides country-wise details of the time series dataset of the two CEs. Each country's time series of collective anxiety and collective positive emotions became the basis of subsequent analysis.

### Identifying structural breaks

Abrupt changes in daily values of both CEs were identified by determining structural breaks. Structural breaks refer to statistically significant, abrupt, and unexpected changes in the values of variables measured at regular time intervals. Scholarly works on structural breaks in the context of econometric data consider them as sources of errors, unless they are explained by an external shock to the series (Chow, 1960; Hansen, 2001; Bai and Perron, 2003). For the purposes of this research, however, structural breaks were valuable sources of information as they indicated the onset of exogenous shocks to collective emotions in a country (Casson and Fry, 2011; Cró and Martins, 2017; Zhang et al., 2017; Guan et al., 2018; Petruželka and Barták, 2020). In other words, structural breaks were statistically significant changes in the levels of anxiety and positive emotions and marked the impact of an event that provided the shock. This property of structural breaks was leveraged in the current project to discover key events of collective emotional significance in context of the pandemic.

The most well-known test for a structural break is the Chow test (Chow, 1960), with a null hypothesis of “no structural change”. The test splits the sample into two periods, and tests the equality of parameters estimated for both periods. However, Chow test requires *a priori* hypothesis of the break point and was not suitable as no hypotheses could be developed about possible significant events causing structural breaks. In an alternative approach to the problem, break dates can be treated as unknown. The method of estimating a single unknown break date (Bai, 1994) has been extended to multiple unknown breaks by Bai (1997a,b) and Bai and Perron (2003). This approach provided the most appropriate way for the identification of the break dates in the context of this study.

R package “strucchange” (Zeileis et al., 2003) was used to implement Bai and Perron's algorithm in the “breakpoints” function to obtain the “break dates” along with their 95% confidence intervals. For each time series, we tested the model


$$\begin{array}{l} Y_{i}=\;X_{i}^{T}\beta_{i}+\mu_{i}, \end{array}$$

with the null hypothesis that regression coefficients remained constant over time. The alternative hypothesis was that at least one coefficient varies over time.


$$\begin{array}{l} H_{0}:\;\beta_{i}=\;\beta_{0}\;\left(i=1,2,\ldots ,n\right). \end{array}$$

F statistics were computed for every variable. The supF test from the “supremum” tests of Andrews (1993) was employed, and *H*_0_ was rejected for statistically significant values. F statistics plots indicating the supF values at the 5% significance level for all series are provided in Supplementary material 3. The number of potential breakpoints was estimated using the Bayesian information criterion (BIC) according to Bai and Perron's recommendation. Supplementary material 3 also provides residual sum of squares (RSSs) and BIC plots.

It is important to note that the research was able to suppress the impact of individual-specific events (such as the death of a loved one) in the estimation of CEs. Anxiety and positive emotion levels in each country were estimated using its *metatext*, which aggregated words from individual messages. Therefore, all detected structural breaks necessarily reflected a statistically significant change in lexical indicators within the sample of Twitter traffic from a country and indicated events that were collectively significant to people from that country. For each structural break, instead of the specific date, the study focused on the period spanning the 95% CI of the break to accommodate the latency of public reaction to events and the spread of countries across the International Date Line. Throughout the study, references to detected structural breaks or shocks pertain to the 95% CI period and not the date itself. For each break discovered in anxiety or positive emotions, global- and country-specific timelines of pandemic-related events were examined.

## Results

### Preliminary results

**Figure 2:**
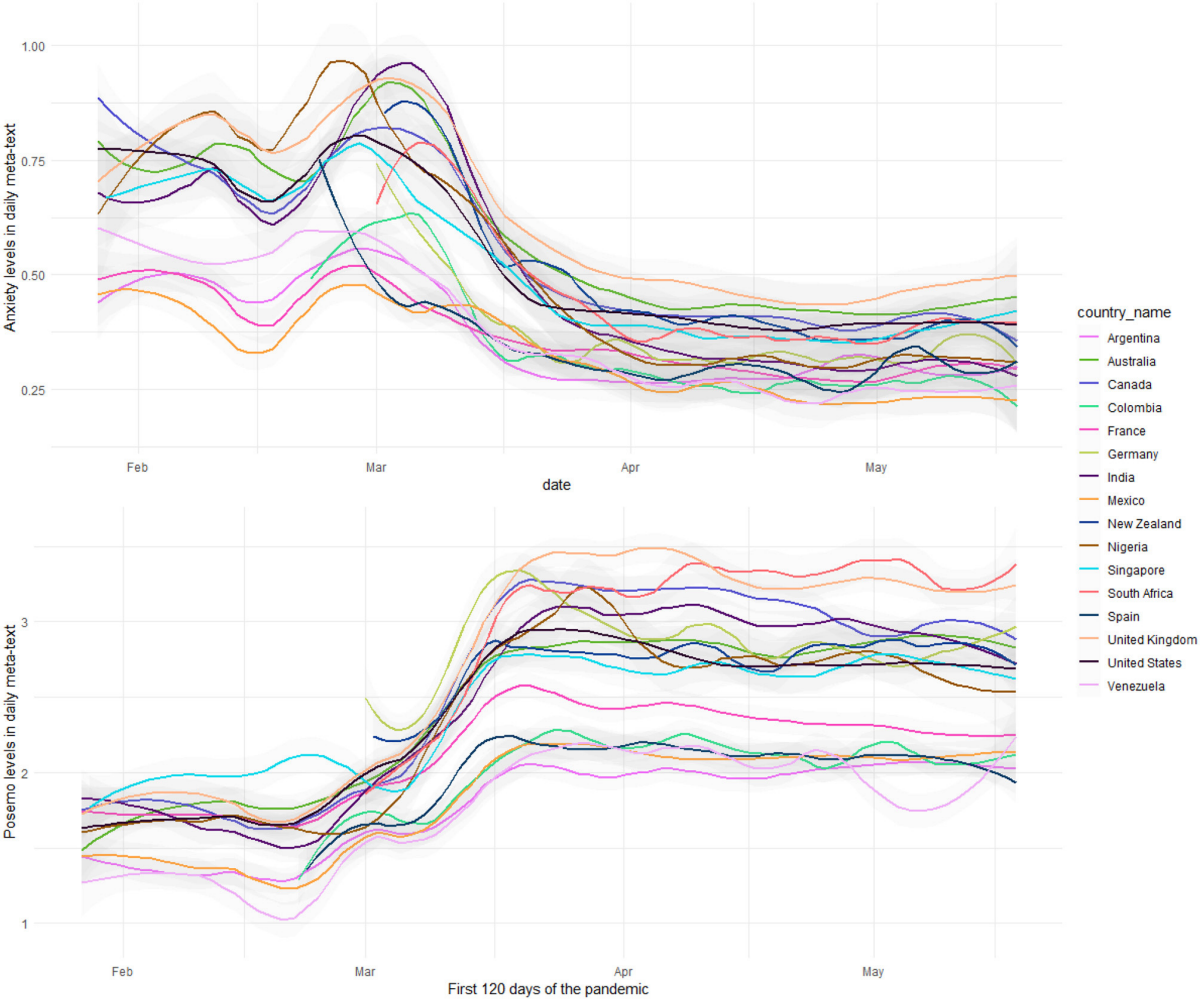
Anxiety and positive emotions in Twitter posts on COVID-19.

The daily *metatext* from all the countries contained a higher proportion of positive emotions lexical indicators than anxiety. During the 120-day period, estimated anxiety was highest in the UK ($\bar{x}=0.63;SD=0.18$) and lowest in Mexico ($\bar{x}=0.33;SD=0.11$); positive emotions were highest in South Africa ($\bar{x}=3.08;SD=0.47$) and lowest in Venezuela ($\bar{x}=1.75;SD=0.42$). Figure 2 presents the time series progression of collective anxiety and collective positive emotions in all the countries.^5^ As the figure captures, consistent with the rapidly changing landscape of the pandemic, both emotions showed considerable volatility until April but became more stable subsequently.

**Figure 3:**
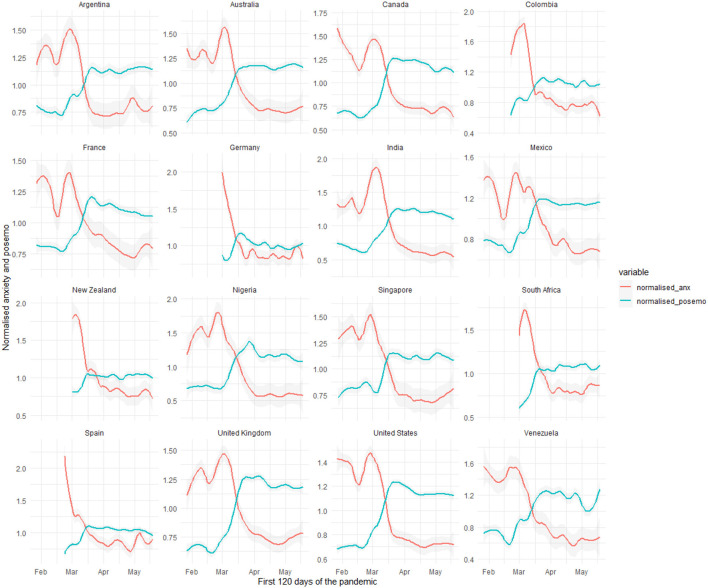
Predominant collective emotion in each count.

In order to determine the predominant CE, the differences in the relative frequency of the lexical indicators were adjusted to obtain a normalized daily score for both anxiety and positive emotions (see Figure 3).^6^ As Figure 3 shows, all the countries had a higher expression of anxiety than positive emotions during January and February. In most countries, the pattern reversed during March and positive emotions dominated over anxiety in COVID-19 posts on Twitter.

### Shocks to CEs identified in each time series

Shocks to anxiety were detected in 15 countries and in total 21 instances of significant and unexpected changes to this CE were detected. Two shocks to anxiety were detected in Australia, Canada, India, New Zealand, South Africa, and Venezuela. France was the only country where no significant shock to anxiety was detected. Shocks to positive emotions were detected in all 16 countries. Three shocks to positive emotions were detected in the USA, two in Germany and India, and one each in the remaining 13 countries giving a total of 20 shocks across all the countries. Tables 2A, B presents the details.

Dates and CIs for all the detected shocks were examined for overlaps and consistency in the direction of change in the values of both CEs. Periods of shocks to anxiety showed a remarkable overlap—in 12 countries, anxiety significantly and abruptly reduced between 9 March and 17 March (see Table 2A). Interestingly, a similar overlap was also observed with positive emotions. In all 16 countries, positive emotions increased significantly and abruptly between 9 March and 17 March (see Table 2B). Looking at the bigger picture revealed by shocks to both collective emotions, the period between 9 and 17 March stands out across all 16 countries in the study. In other words, the data suggest that the period saw events of global significance within the context of COVID-19—events that altered the levels of collective emotions in all the countries examined in this research. The global and country-specific timelines for COVID-19 related events were examined to identify the likely sources of shocks to the two collective emotions. One global event and two thematic categories of events appear to be the appropriate explanation for an abrupt increase in collective positive emotions and decrease in collective anxiety. There are discussed next.

**Table 2A:** Break dates in anxiety, CIs, and segment means.

	**Structural breaks in anxiety, their dates, and CIs**		
**Country**	**Break 1 (date and 95% CI)**	**Break 2 (date and 95% CI)**	**Mean and SD of segments created by structural breaks**
Argentina^*^	11 Mar; 10–14 Mar	-	[0.49, 0.11]; [0.29, 0.04]
Australia^*^	10 Mar; 9–14 Mar	29 Mar; 28 Mar−3 Apr	[0.78, 0.16]; [0.46, 0.08]; [0.43, 0.03]
Canada^*^	11 Mar; 10–12 Mar	27 Mar; 26–31 Mar	[0.76, 0.11]–[0.43, 0.06]–[0.40, 0.03]
Colombia^*^	11 Mar; 10–12 Mar	-	[0.58, 0.07]; [0.28, 0.05]
France	-	-	-
Germany^*^	10 Mar; 9–13 Mar	-	[0.58, 0.11]; [0.34, 0.05]
India^*^	12 Mar; 11–15 Mar	28 Mar; 27 Mar−2 Apr	[0.75, 0.20]; [0.36, 0.10]; [0.31, 0.03]
Mexico	1 Apr; 29 Mar−3 Apr	-	[0.40, 0.10]; [0.24, 0.03]
New Zealand^*^	12 Mar; 11–16 Mar	24 Mar; 22–26 Mar	[0.83, 0.17]; [0.49, 0.08]; [0.39, 0.05]
Nigeria	27 Mar; 26–31 Mar	-	[0.73, 0.24]; [0.31, 0.04]
Singapore^*^	22 Mar; 21–27 Mar	-	[0.66, 0.15]; [0.38, 0.06]
South Africa^*^	13 Mar; 12–16 Mar	25 Mar; 24–27 Mar	[0.75, 0.11]; [0.44, 0.08]; [0.37, 0.04]
Spain^*^	17 Mar; 15–19 Mar	-	[0.48, 0.13]–[0.29, 0.04]
United Kingdom^*^	10 Mar; 9–12 Mar	-	[0.83, 0.12]–[0.50, 0.08]–[0.47, 0.04]
United States^*^	11 Mar; 10–13 Mar	-	[0.74, 0.09]–[0.41, 0.04]
Venezuela^*^	12 Mar; 11–16 Mar	31 Mar; 28 Mar−3 Apr	[0.55, 0.13]–[0.27, 0.06]–[0.25, 0.04]

^*^Countries with overlapping periods of shock. Shocks detected in 13 countries between 9 and 17 March.

**Table 2B:** Break dates in positive emotions, CIs, and segment means.

	**Structural breaks in positive emotions, their dates, and CIs**			
**Country**	**Break 1 (date and 95% CI)**	**Break 2 (date and 95% CI)**	**Break 3 (date and 95% CI)**	**Mean and SD of segments created by structural breaks**
Argentina^*^	12 Mar; 11–14 Mar	-	-	[1.43, 0.22]–[2.02, 0.10]
Australia^*^	11 Mar; 10–12 Mar	-	-	[1.84, 0.26]–[2.84, 0.13]
Canada	12 Mar; 11–13 Mar	-	-	[1.83, 0.23]–[3.12, 0.16]
Colombia^*^	11 Mar; 10–13 Mar	-	-	[1.63, 0.19]–[2.14, 0.13]
France^*^	11 Mar; 10–12 Mar	-	-	[1.77, 0.20]–[2.38, 0.13]
Germany^*^	11 Mar; 10–12 Mar	29 Mar; 26 Mar–1 Apr	-	[2.31, 0.22]–[3.01, 0.23]
India	1 Mar; 27 Feb−2 Mar	17 Mar; 16–19 Mar	-	[1.67, 0.19]–[2.81, 0.41]–[2.99, 0.16]
Mexico^*^	12 Mar; 11–14 Mar	-	-	[1.43, 0.23]–[2.12, 0.12]
New Zealand^*^	12 Mar; 11–14 Mar	-	-	[2.27, 0.21]–[2.81, 0.15]
Nigeria^*^	10 Mar; 9–11 Mar	-	-	[1.69, 0.29]–[2.79, 0.32]
Singapore^*^	10 Mar; 9–11 Mar	-	-	[1.97, 0.20]–[2.70, 0.18]
South Africa^*^	12 Mar; 11–13 Mar	-	-	[2.10, 0.22]–[3.26, 0.22]
Spain^*^	12 Mar; 11–14 Mar	-	-	[1.66, 0.20]–[2.13, 0.10]
United Kingdom^*^	12 Mar; 11–13 Mar	-	-	[1.91, 0.26]–[3.30, 0.19]
United States^*^	25 Feb; 23–26 Feb	12 Mar; 11–13 Mar	8 Apr; 7–10 Apr	[1.68, 0.10]–[2.60, 0.39]– [2.78, 0.11]– [2.71, 0.05]
Venezuela^*^	13 Mar; 11–15 Mar	-	-	[1.33, 0.24]–[2.05, 0.24]

^*^Countries with overlapping periods of shock. Shocks detected in all 16 countries between 9 and 17 March.

#### WHO's declaration of the pandemic had a positive emotional impact in all countries

Between 9 and 17 March, an external shock to collective positive emotions was observed in all countries, and to anxiety in 12 countries (see Tables 2A, B). 28 out of the total 41 shocks (68%) detected in both CEs across all countries falling within this narrow period of nine days strongly points in the direction of an event of global relevance.

The directions of changes in the emotions were also consistent—anxiety declined, and positive emotions increased after the shock event. Within this period, the only event that cut across national boundaries and held relevance for people across the world was the WHO's declaration on 11 March where it formally assigned COVID-19 the label of a pandemic. Indeed, the 95% CI estimates for the shock date include the 11 March in all the countries for at least one CE, and in nine countries, for both CEs (see Tables 2A, B). Thus, the current research suggests that WHO's declaration of pandemic was an emotionally significant event globally where it led to an increase in collective positive emotions and decrease in collective anxiety.

#### Lockdowns and declarations of emergencies had a positive emotional impact

The periods of shocks in nine countries-when anxiety abruptly declined and positive emotions grew-coincided with announcements of national health emergencies, restrictions on civil liberties, and lockdowns. Four countries made these announcements very close to the WHO's declaration of a pandemic. These included Colombia and the USA, where shocks to both emotions coincided with the declaration of emergencies on 12 and 13 March, respectively. In Germany, periods of shocks to both emotions coincided with Chancellor Angela Merkel's first press conference on the situation on 11 March, where she announced a general ban on large public events. Finally, Venezuela's stay-at-home order on 15 March overlapped with shocks detected in both emotions in the country.

In a further five countries, periods of shocks to anxiety coincided with similar measures later in March. In Mexico, it coincided with the declaration of the national health emergency on 30 March, in New Zealand, with a national lockdown on 25 March, and in Nigeria and Spain with restrictions becoming more stringent. Finally, of the two shocks to anxiety detected in South Africa, one coincided with a declaration of a “national state of disaster” on 15 March, while the second was only a day removed from the announcement of a national lockdown on 23 March.

To summarize, announcements of national restrictions and lockdowns were the common denominators of a decline in anxiety or a rise in positive emotions in nine countries. A total of 13 breaks detected in the data can be explained by these announcements acting as an exogenous shock.

#### Announcement of economic support packages had a positive emotional impact

Eight shocks to emotions in five countries coincided with the announcement of COVID-19-specific economic support packages in the respective countries. In each instance, they were marked by a reduction in anxiety or an increase in positive emotions. For Australia, the detected shock to anxiety and positive emotions overlapped with the governmental announcements of economic support packages on 12 March and 30 March. In Canada, along with the WHO's declaration of a pandemic, the periods of shock to positive emotions coincided with the government's economic package announcements on 11 and 13 March. Finally, in Singapore, the detected shock and reduction in anxiety coincided with the announcement of the coronavirus resilience budget on 26 March.

#### No clear exogeneous shocks

Seven breaks in six countries did not map out concretely with any local or global events that could have explained the shocks to the series. These include two positive emotions breaks in the USA (25 February; CI: 23–26 February and 8 April; 7–10 April) and one in India (1 March; CI: 27 February−2 March). Anxiety breaks in Canada (27 March; CI: 26–31 March), Peru (7 March; CI: period of shock: 5–9 March), and Venezuela (31 March; CI: 28 March−3 April) also did not have any clear events that coincided with the breaks. Finally, in one instance positive emotions dropped following a detected break in the time series (USA, break on 8 April; CI: 7 April−10 April). While no significant national event could be identified, it is worth noting that the break was observed a day after President Donald Trump's unsubstantiated criticism of the WHO on Twitter on 7 April.

## Discussion

The work presented in this study is among the first to combine big-data research, natural language processing techniques, and time series analysis to examine collective psychological phenomena. It is also among the first to examine collective emotions in the context of a global phenomenon and study them during the same time period in multiple countries. To the best of the authors' knowledge, this research is the first reported work to move away from survey methodologies to identify significant emotional events within and across countries by using naturally occurring text on Twitter. These methodological developments and the practical application of collective emotions were one of the main motivations for sharing this work with our peers.

Due to resource constraints, the current study did not examine the text of the Twitter posts beyond using LIWC to identify lexical indicators for anxiety and positive emotions. Future studies could, for example, also use topic modeling to scaffold the identification of the main topics as a way of validating the events that explain the shock. Due to the large number of data points and individual posters in the dataset, we did not filter for bots. However, the texts posted by bots and messages with false rumors and misinformation are no different in their contributions to the development of collective emotions.

Findings of this research have implications for public policy during periods of disasters and national crisis. As reported, in five countries, the announcement of economic support packages by their respective governments helped reduce anxiety and enhance positive emotions amidst the pandemic. It is likely that the announcement of economic support packages allowed people to come to terms with job losses and other financial challenges posed by the pandemic. This finding emphasizes the significance of providing economic security to the public during times of unprecedented uncertainty and disruption. A very important finding from this project is regarding the impact of the WHO's declaration of a pandemic on public emotions. The WHO was heavily criticized for declaring H1N1 swine flu as a pandemic in 2009 (Godlee, 2010; Low and McGeer, 2010), but with COVID-19, its reluctance in using the term received significant attention (Green, 2020; Vogel, 2020). In part, the reluctance reflected the conventional wisdom that terms such as “pandemic” and “epidemic” cause or exacerbate fear and panic among the public (Van Damme and Van Lerberghe, 2000; Bonneux and Van Damme, 2006; Barrett and Brown, 2008; Honigsbaum, 2009; Arafat et al., 2020; Maxmen, 2021). The WHO director Tedros Adhanom Ghebreyesus explicitly shared this concern in his press conference on 23 February 2020, where he observed that “using the word pandemic [now] does not fit the facts, but it may certainly cause fear” (WHO, 2020). Fear has an adaptive role in preventing people from harm, and researchers have previously noted that apprehensions about mass panic triggered by disasters and emergencies are exaggerated (Quarantelli, 2001; Gantt and Gantt, 2012; Van Bavel et al., 2020). In this light, the present research adds to the evidence base—instead of reacting with heightened anxiety, people in the 16 countries showed an increase in positive emotions and a decline in anxiety, and around the middle of March, anxiety ceased to be the predominant CE in all countries. This partially follows a natural decay in anxiety, as other studies have also reported (Mertens et al., 2023). However, results from time series analysis undertaken in this research provide strong evidence that the WHO's declaration of the pandemic was likely to have contributed to the decline in anxiety. It is well understood that uncertainty reduction in ambiguous situations lowers anxiety (Reuman et al., 2015; Chen et al., 2018) following evolutionary (Brosschot et al., 2016) and neurobiological (Grupe and Nitschke, 2013) pathways, and one possible explanation is that the WHO's declaration reduced uncertainty and confusion around the threat posed by the novel disease. Finally, this study also provided evidence that national lockdowns, declarations of states of emergency, and announcements of social restrictions did not result in a negative emotional reaction in any country. In none of the countries these measures coincided with an increase in anxiety or decline in positive emotions. On the contrary, Twitter posts in nine countries showed either a reduction in anxiety or an increase in positive emotions following such announcements, suggesting a largely positive reception of these measures in most countries. In this regard, the current study further emphasizes the need to interrogate assumptions around mass panic in the public sphere during periods of disaster and emergency.

## Data availability statement

Publicly available datasets were analyzed in this study. This data can be found here: https://zenodo.org/record/7213964.

## Author contributions

AC, SS, and VB conceptualized the project and were involved in all the stages of the project including planning, data generation, cleaning, analysis, and preparation of the manuscript. AC and VB led the data analysis stage of the project. AC and SS led the writing of the manuscript. HS and JP led the data cleaning and LIWC processing of the dataset. AC led the project overall. All authors have approved the manuscript.

## Footnotes

^1^As a common global event early in the pandemic, the confirmation of human-to-human transmission of the virus on 20 January 2020 was taken as day 1 of the pandemic.

^2^Documenting the Now. (2020). Hydrator [Computer Software]. Retrieved from https://github.com/docnow/hydrator.

^3^New Zealand, with its uninterrupted time series starting on 2nd March, was included as an exception due to its theoretical value as a country with unusually low COVID-19 cases during the period of the study.

^4^LIWC's validated dictionaries of English, French, and Spanish were used according to the predominant language of the country. It is expected that some Twitter posts in data from all countries were made in a language other than the one identified as its main language. However, given the size of the dataset, this would have been a relatively small proportion, as indicated by high lexicon hit rates observed for all the countries ($\bar{x}=69.75,\;SD=5.02$).

^5^Smoothened for better visual presentation using LOESS (Cleveland et al., 2017). The parameter α was set to a period of 21 days closest to each data point.

^6^This was achieved by dividing each observation in each series by the respective mean of the series *X*_*i*_(*t*) = *X*_*i*_(*t*)/μ(*t*).
