# Supplementary material for: Tracking collective emotions in 16 countries during COVID-19: a novel methodology for identifying major emotional events using Twitter
Source: Front Psychol. 2024 Mar 25;14:1105875. doi: 10.3389/fpsyg.2023.1105875 (PMC11000126; doi:10.3389/fpsyg.2023.1105875)
Supplement: Supplementary file 1 [file Data_Sheet_1.PDF]

<u>SUPPLEMENTARY MATERIAL 1:</u> Details of data preparation and cleaning

We used Python software for this work. Codes for this work are available in the uploaded folder "Data Preparation Codes and Scripts"

Batch Division and Rehydration of tweet IDs obtained from the dataset: We used the original cleaned twitter dataset from Zenodo Version 7.0 (https://zenodo.org/record/3749360), uploaded on 26/04/2020 for tweet ids for January 1 – April 25. For April 26 – May 18, versions 8.0, 9.0, 10.0, and 11.0 were used. The original dataset contained index, date_time and tweet IDs. We used rehydratoR Application (version 0.0.5) and extracted the content of the twitter message. The application returned the corresponding tweets associated with the IDs along with other information like date, retweet count, location etc. in the form of JSON objects. 9 per cent tweets in the dataset had been either deleted or made unavailable by people who posted it and were no longer publicly available.

Using JSON objects extract details of twitter messages: We parsed through the json files and extracted the required columns [Created_at, month, date, time, day, tweet_id, country, location, full_text, language, retweet count, favourite_count, reply_count] from each object. We had identified 25 countries with a large number of users on Twitter (see methods) and if the "country" column matched the name of the country, the tweet was placed in the data for that country. If the tweet country was missing, we looked at the location column and extracted single individual tokens from it and checked if it was present in our list of regions, states, and towns and cities for countries available at [https://www.diva-gis.org/gdata] which is based on the

GADM dataset version 2.5 from July 2015. If a match was found, the tweet got placed in the dataset of that country. For example, if a twitter message did not have anything in the country column but had "New York" in the location column, the tweet was placed in the USA dataset. Following this process, all the tweets in JSON files were parsed and the ones belonging to any of our target countries were picked and placed in the right dataset. This country wise dataset is further split up by date to create the final dataset.

Text File Generation: Once the csv files were ready, we converted them into LIWC compatible text files for each date. To achieve this we traversed through the csv file and appended the tweet text to the text file for that particular date – all the tweets for a particular date were combined into a single text file. Emojis were not removed as LIWC does not process them as words. We removed URLs that were inserted by some users in their Twitter posts and performed data cleaning to ensure consistent encoding. As a result, we had a folder for each country, containing around 120 text files, one for each date. This folder was passed as input to LIWC and it performed linguistic analysis on these files, giving us date wise output for each country.

Supplementary Material 2: Original and Imputed values across countries

We used R software for this work. Codes for this work are available in the uploaded folder “Data and scripts for main analysis and figures”

We screened the date wise meta-text for each country using the threshold of 5,000 words (see Python script 6). The dataset used in the project had a systematic error and had a very low number of tweets on four dates (08/02/2020; 09/02/2020; 28/02/2020; 29/02/2020). The dates affected all countries in the sample. 16/03/2020 was another date when the number of tweets were systematically low which meant that seven smaller countries in the sample fell below 5000-word limit and had to be imputed. Beyond these known and systematic dates, Australia, Colombia, New Zealand, and Singapore required imputation for one date each.

Imputation was carried out separately for each country. For entire data of all countries consisting of 1918 rows, only 75 rows were inputted (that is, 3.91% of the data). The R package Amelia II was used for imputation. It uses EM algorithm for imputing missing data. It can be used for imputation in time-series data. R code used for imputation is provided with the submission.

The following tables provide details of the imputation process:

- Table 2.1: Imputed dates in the data
- Table 2.2: Imputation details for each country
- Table 2.3: Original and imputed data values

Table 2.1: Imputed dates in the data (those marked with asterisk are systematic errors in dataset)

Sr.	Date	Number of Countries < 5000 WC	Counties (Three letter code)
**1**	08/02/20*	8	AUS, CAN, FRA, SGP, GBR, VEN, MEX, NGA
**2**	09/02/20*	11	ARG, AUS, CAN, FRA, IND, SGP, GBR, USA, VEN, MEX, NGA
**3**	10/02/20	1	AUS
**4**	22/02/20	1	SGP
**5**	28/02/20*	12	ARG, AUS, CAN, COL, FRA, IND, SGP, GBR, VEN, MEX, ESP, NGA
**6**	29/02/20*	12	ARG, AUS, CAN, COL, FRA, IND, SGP, GBR, VEN, MEX, ESP, NGA
**7**	01/03/20	1	COL
**8**	03/03/20	1	NZL
**9**	16/03/20	7	FRA, DEU, SGP, NZL, ESP, NGA, ZAF

Table 2.2: Imputation details for each country (those marked with asterisk are systematic errors in dataset)

Country	Number of observations in a series	Number of dates for which data was imputed	% of imputed data points
**ARG ***	113	3	2.65
**AUS ***	113	5	4.42
**CAN ***	113	4	3.54
**COL**	87	3	3.45
**DEU ***	79	1	1.26
**ESP ***	86	3	3.49
**FRA ***	113	5	4.42
**GBR ***	113	4	3.54
**IND ***	113	3	2.65
**MEX ***	113	4	3.54
**NGA ***	113	5	4.42
**NZL**	78	2	2.56
**SGP**	112	6	5.36
**USA ***	113	1	0.88
**VEN ***	113	4	3.54
**ZAF ***	79	1	1.27

**TOTAL**	**1651**	**54**	
**Average**	**103.19**	**3.375**	**3.67**

Table 2.3: Original and imputed data values

			Imputed values			Original values		
**Country Name**	**Code**	**Date**	**WC**	**Posemo**	**Anx**	**WC**	**Posemo**	**Anx**
Argentina	ARG	09/02/20	15895.50	1.4131	0.5111	96	4.17	0.00
Argentina	ARG	28/02/20	39679.75	1.5748	0.4423	526	1.52	0.95
Argentina	ARG	29/02/20	39679.75	1.5543	0.4286	231	0.87	0.43
Australia	AUS	08/02/20	12870.00	1.8866	0.7429	3759	1.70	0.53
Australia	AUS	09/02/20	12272.00	1.9104	0.7535	33	6.06	0.00
Australia	AUS	10/02/20	11319.25	1.8882	0.7585	3419	1.73	0.73
Australia	AUS	28/02/20	16507.50	1.8322	0.7683	251	0.80	1.20
Australia	AUS	29/02/20	18497.38	2.0037	0.7231	179	2.23	0.56
Canada	CAN	08/02/20	16205.25	1.7670	0.7804	4158	1.68	0.48
Canada	CAN	09/02/20	16943.31	1.8069	0.7693	253	0.40	0.40
Canada	CAN	28/02/20	16546.50	1.8391	0.7531	868	2.07	0.92
Canada	CAN	29/02/20	15972.63	1.8611	0.7246	455	1.54	0.44
Colombia	COL	28/02/20	11392.25	1.6329	0.5673	280	1.07	1.07
Colombia	COL	29/02/20	8224.81	1.6054	0.5850	75	4.00	0.00
Colombia	COL	01/03/20	9779.27	1.6249	0.5791	4260	1.92	0.56
France	FRA	08/02/20	11746.75	1.7424	0.4506	987	1.42	0.10

France	FRA	09/02/20	12613.94	1.7302	0.4503	182	1.65	0.00
France	FRA	28/02/20	29271.75	1.7466	0.4590	760	1.71	0.79
France	FRA	29/02/20	29334.44	1.8345	0.4701	826	2.06	0.12
France	FRA	16/03/20	64110.25	2.2750	0.4195	1198	2.00	0.08
Germany	DEU	16/03/20	28574.50	2.9538	0.4386	534	1.12	0.37
India	IND	09/02/20	28392.50	1.7691	0.7336	208	1.44	0.48
India	IND	28/02/20	37505.00	1.8005	0.7669	595	1.01	1.68
India	IND	29/02/20	57770.75	1.7647	0.7918	529	2.08	1.51
Singapore	SGP	08/02/20	11991.50	2.1887	0.6559	4585	1.55	0.74
Singapore	SGP	09/02/20	13317.88	2.1914	0.6779	95	3.16	0.00
Singapore	SGP	22/02/20	8314.00	2.1682	0.6385	4933	1.66	0.85
Singapore	SGP	28/02/20	7409.25	2.0907	0.6779	91	2.20	2.20
Singapore	SGP	29/02/20	8090.56	2.2052	0.6553	50	6.00	0.00
Singapore	SGP	16/03/20	9831.25	2.4671	0.5831	1281	3.51	0.70
United Kingdom	GBR	08/02/20	35857.75	1.8359	0.8495	3413	1.61	0.91
United Kingdom	GBR	09/02/20	38179.69	1.9847	0.8093	249	4.02	0.00
United Kingdom	GBR	28/02/20	50096.00	1.9809	0.8178	1401	1.64	1.14
United Kingdom	GBR	29/02/20	57058.75	2.0227	0.7842	926	2.16	0.43
United States	USA	09/02/20	130395.25	1.7654	0.7446	1459	1.64	0.55
Venezuela	VEN	08/02/20	14858.50	1.2972	0.5341	2987	1.14	0.64
Venezuela	VEN	09/02/20	15003.88	1.2992	0.5738	70	0.00	0.00
Venezuela	VEN	28/02/20	21745.75	1.3994	0.5572	819	1.47	0.49
Venezuela	VEN	29/02/20	16620.69	1.2916	0.5429	610	2.46	0.00
New Zealand	NZL	03/03/20	7752.33	2.4762	0.7278	4422	2.24	1.09
New Zealand	NZL	16/03/20	13767.25	2.6203	0.6519	1899	2.95	0.32
Mexico	MEX	08/02/20	12814.75	1.3669	0.4219	4491	1.54	0.42
Mexico	MEX	09/02/20	13583.69	1.4768	0.4039	157	1.91	0.00

Mexico	MEX	28/02/20	19586.25	1.5527	0.4367	1592	1.76	0.82
Mexico	MEX	29/02/20	17626.06	1.5486	0.3945	782	2.17	0.26
Spain	ESP	28/02/20	16699.50	1.6312	0.5181	254	0.39	0.00
Spain	ESP	29/02/20	12952.38	1.5909	0.5050	161	1.24	0.00
Spain	ESP	16/03/20	44928.75	2.2333	0.3746	1199	1.92	0.50
Nigeria	NGA	08/02/20	12811.25	1.8656	0.7873	1667	1.68	0.66
Nigeria	NGA	09/02/20	12623.31	2.0227	0.7871	71	1.41	1.41
Nigeria	NGA	28/02/20	23931.00	1.8964	0.7734	2732	1.90	1.06
Nigeria	NGA	29/02/20	25972.25	2.0092	0.7306	1063	0.94	1.13
Nigeria	NGA	16/03/20	49033.25	2.0539	0.7730	751	1.73	0.93
South Africa	ZAF	16/03/20	25526	2.7908	0.5843	450	3.56	0.22

Note: Posemo = Positive Emotions; Anx = Anxiety

We used R software for this work. Codes for this work are available in the uploaded folder "Data and scripts for main analysis and figures"

<u>Supplementary Material 3: Decisions on number of structural breaks</u>

This section presents two figures per country per variable.

There are 32 (16 countries X 2 variable) figures. Each of the 38 figures has four graphs: The four graphs are as follows: (A) The BIC and RSS values over number of breakpoints; (B) Distribution of F statistics for SupF test; (C) BIC suggested Breaks and confidence intervals; and (D) Plot of Structural Break with segment averages for finally retained breaks.

These graphs explain decision-making regarding the number of structural breaks Figure D presents the final solution retained for that variable for a given country. The details are as follows:

(A) The BIC and RSS values over number of breakpoints: This figure shows BIC and RSS values over possible breakpoints starting from 0 to 5 or 6

(B) Distribution of F statistics for SupF test: This figure shows distribution of the F statistics and the horizontal red line shows the significant F value.

(C) BIC suggested Breaks and confidence intervals (CI): This figure plots all BIC suggested breaks with the breakpoints. The 95% CI for each break is plotted by a red vertical line at bottom of the break. The break date/s is/are provided below the graph and the confidence interval (in days) is given in parenthesis.

(D) Plot of Structural Break with segment averages for finally retained breaks: This plot shows finally retained breaks. The red vertical line is break-date. The green horizontal line shows the average of each segment created by the break. The grey

dotted line is the average of the entire series. The break-date/s and segment-wise averages are provided below the plot.

Argentina:

Figure 1: Plots for Positive Emotion - Argentina

(A) The BIC and RSS values over number of breakpoints

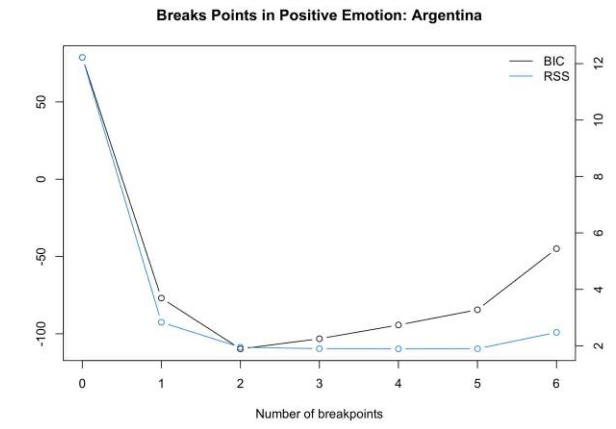

(B) Distribution of F statistics for SupF test.

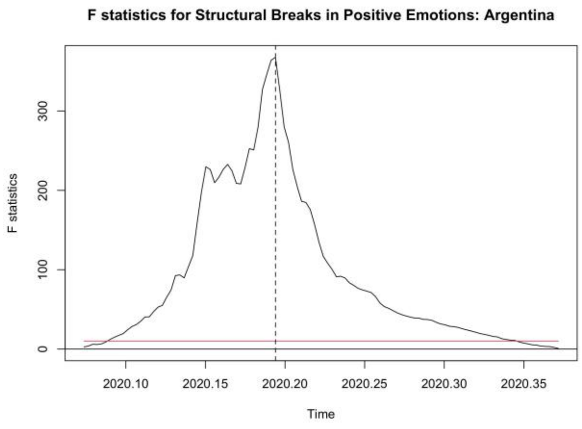

(C) BIC suggested Breaks and confidence intervals

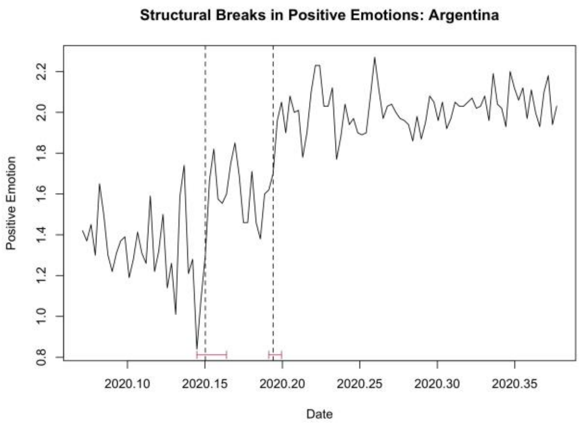

BIC suggested two breaks on 25/02/2020 (CI = 7) and 12/03/2020 (CI = 3)

(D) Plot of Structural Break with segment averages for finally retained breaks.

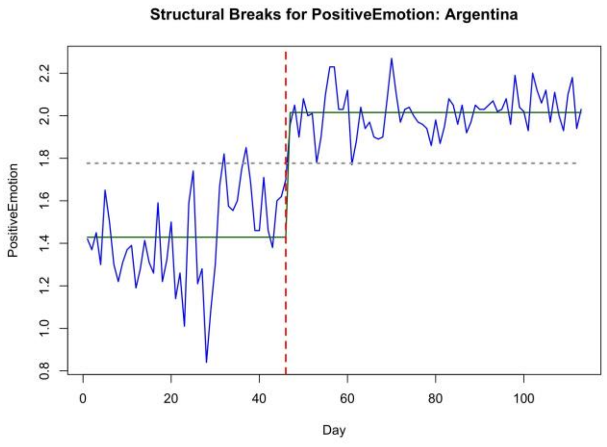

Breaks date = 12/03/2020

Segment averages: Segment1 = 1.43; Segment 2 = 2.02

Figure 2: Plots for Anxiety - Argentina

(A) The BIC and RSS values over number of breakpoints

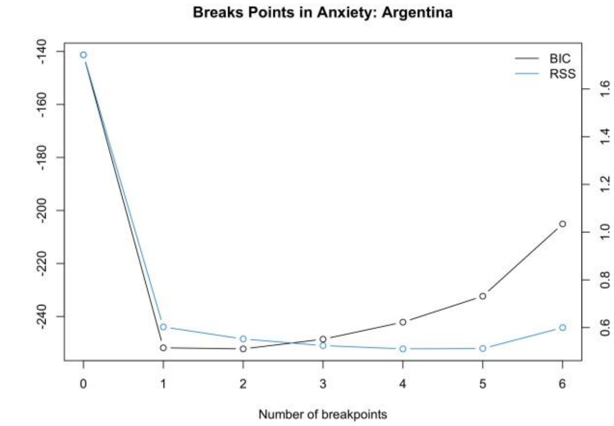

(B) Distribution of F statistics for SupF test.

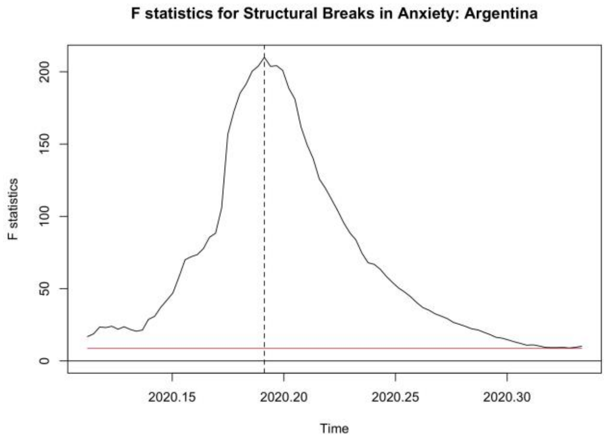

(C) BIC suggested Breaks and Confidence Intervals

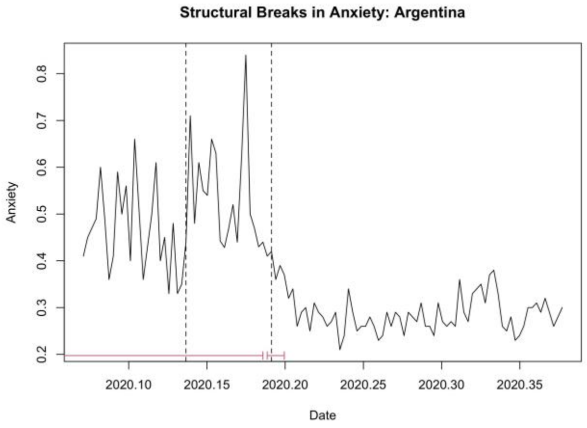

BIC suggested two breaks on 20/02/2020 (CI = 50) and 11/03/2020 (CI = 4)

(D) Plot of Structural Break with segment averages for finally retained breaks.

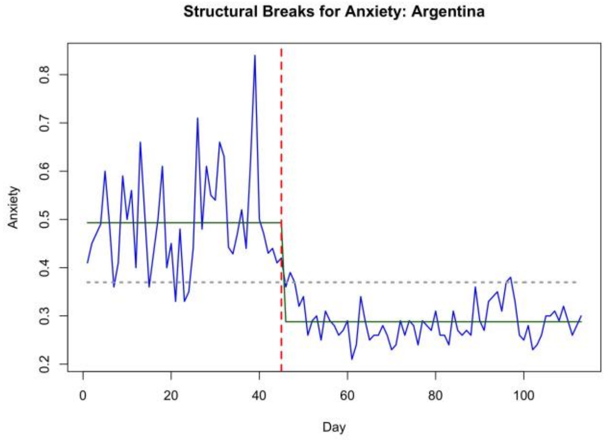

Breaks date = 11/03/2020

Segment averages: Segment1 = 0.49; Segment 2 = 0.29

Australia

Figure 3: Plots for Positive Emotion – Australia

(A) The BIC and RSS values over number of breakpoints

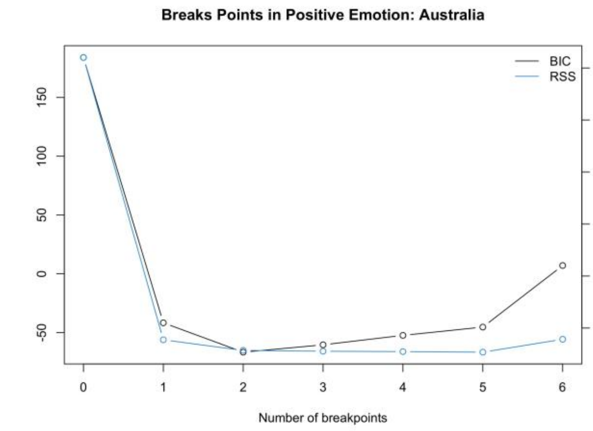

(B) Distribution of F statistics for SupF test.

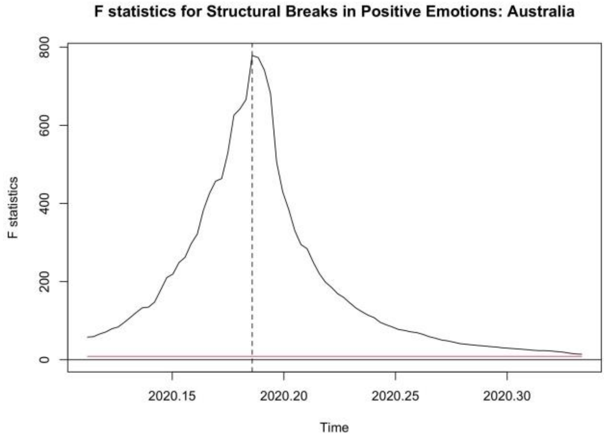

(C) BIC suggested Breaks with Confidence Interval.

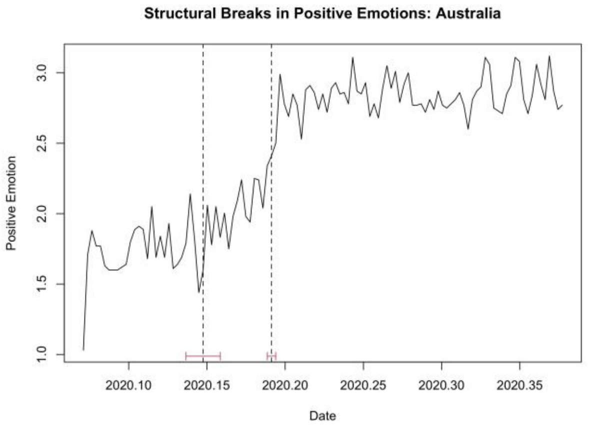

BIC suggested two breaks on 24/02/2020 (CI = 8) and 11/03/2020 (CI = 2)

(D) Plot of Structural Break with segment averages for finally retained breaks.

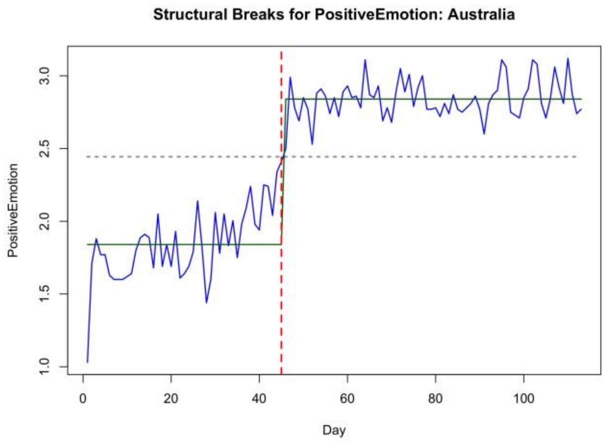

Breaks date = 11/03/2020

Segment averages: Segment1 = 1.84; Segment 2 = 2.84

Figure 4: Plots for Anxiety - Australia

(A) The BIC and RSS values over number of breakpoints

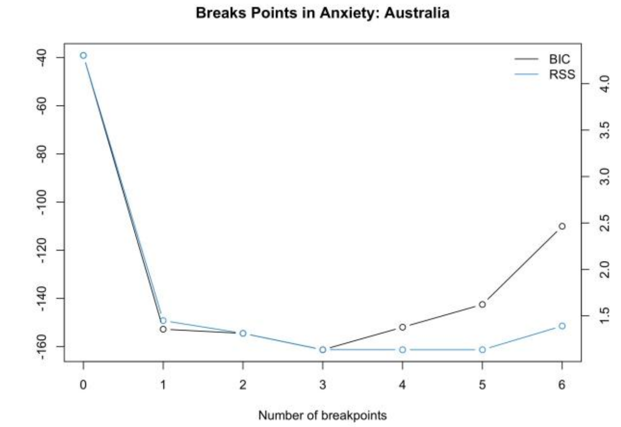

(B) Distribution of F statistics for SupF test.

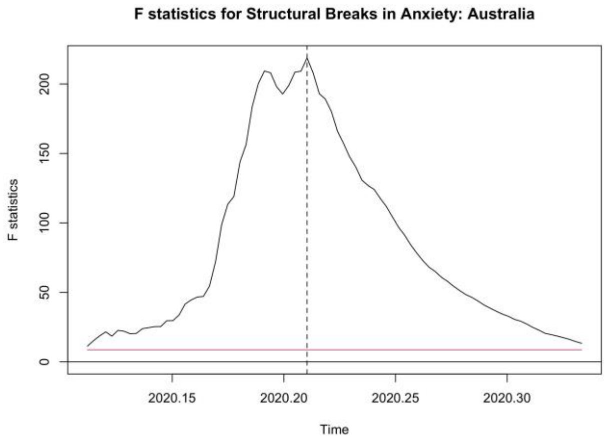

(C) BIC suggested Breaks with Confidence Interval.

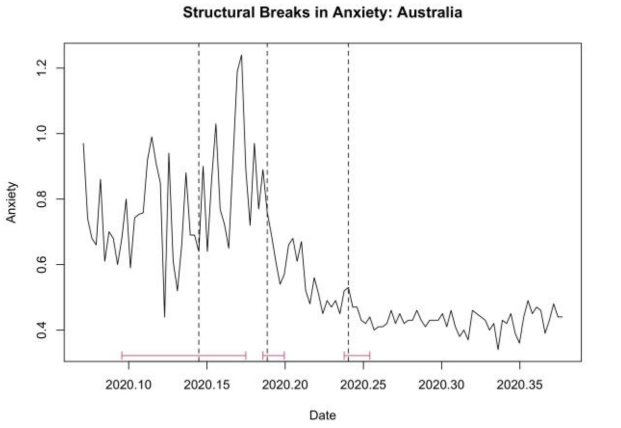

BIC suggested three breaks on 23/02/2020 (CI = 29) and 10/03/2020 (CI = 05) and 29/03/2020 (CI = 6)

(D) Plot of Structural Break with segment averages for finally retained breaks.

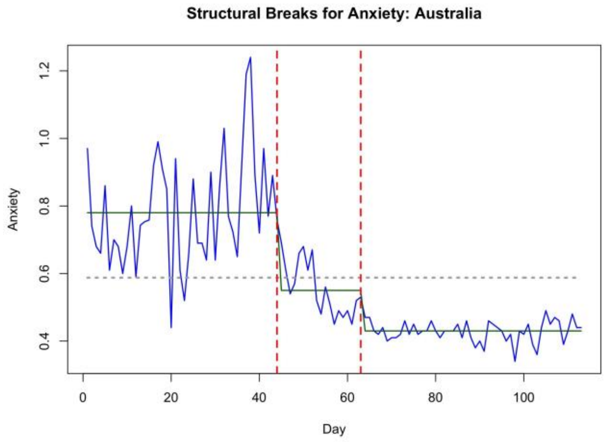

Breaks date = 10/03/2020 and 29/03/2020

Segment averages: Segment1 = 0.78; Segment 2 = 0.55; Segment3 = 0.43.

Canada

Figure 7: Plots for Positive Emotion – Canada

(A) The BIC and RSS values over number of breakpoints

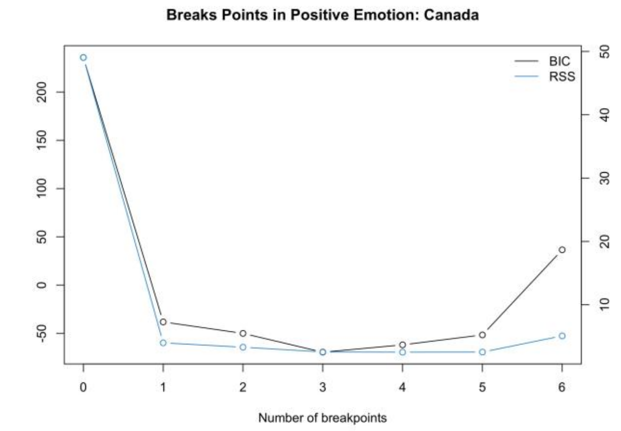

(B) Distribution of F statistics for SupF test.

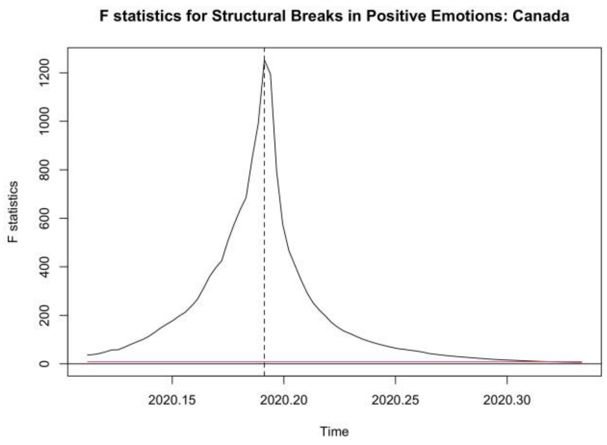

(C) BIC suggested Breaks and confidence interval

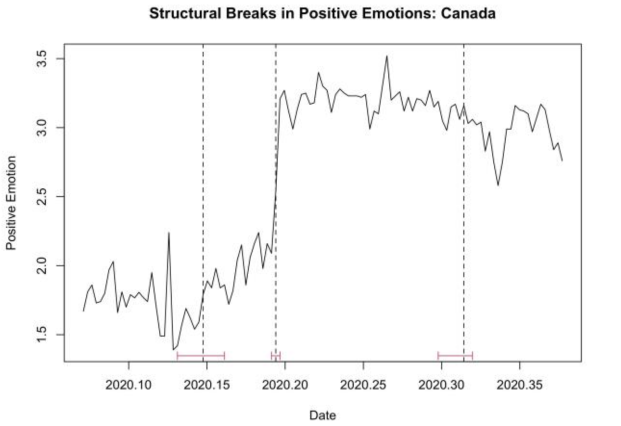

BIC suggested three breaks on 24/02/2020 (CI = 11) and 12/03/2020 (CI = 2) and 25/04/2020 (CI = 8)

(D) Plot of Structural Break with segment averages for finally retained breaks.

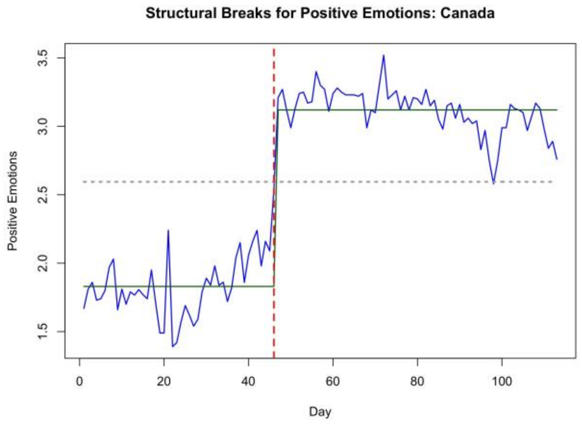

Breaks date = 12/03/2020

Segment averages: Segment1 = 1.83; Segment 2 = 3.12

Figure 8: Plots for Anxiety - Canada

(A) The BIC and RSS values over number of breakpoints

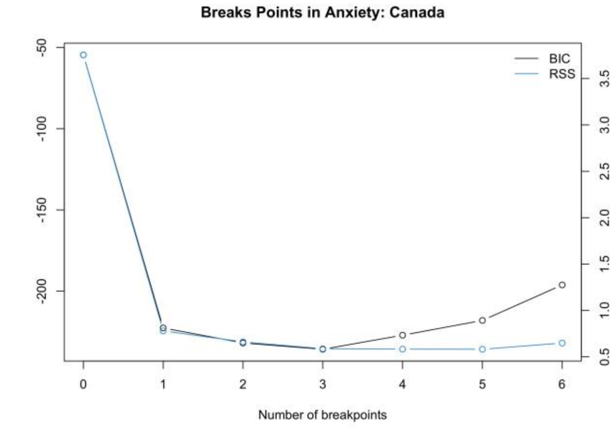

(B) Distribution of F statistics for SupF test.

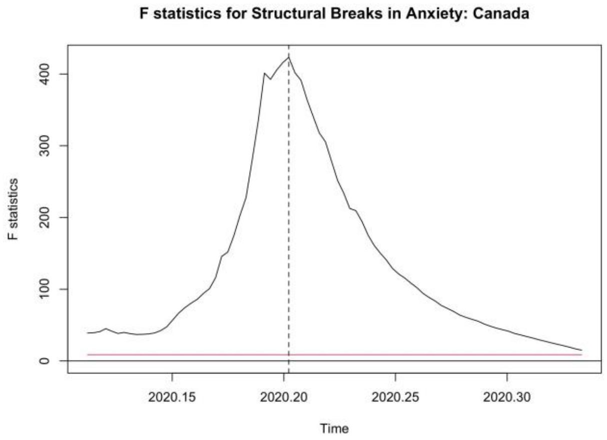

(C) BIC suggested Breaks with Confidence Interval

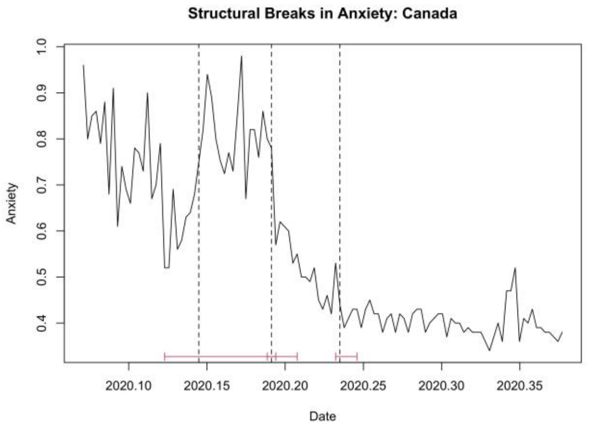

BIC suggested three breaks on 23/02/2020 (CI = 31) and 11/03/2020 (CI = 2) and 27/03/2020 (CI = 5)

(D) Plot of Structural Break with segment averages for finally retained breaks

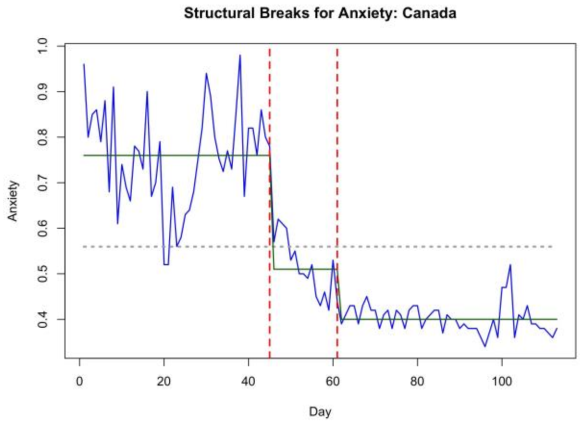

Breaks date = 11/03/2020 and 27/03/2020

Segment averages: Segment1 = 0.76; Segment 2 = 0.51; Segment3 = 0.40.

Colombia

Figure 11: Plots for Positive Emotion – Colombia

(A) The BIC and RSS values over number of breakpoints

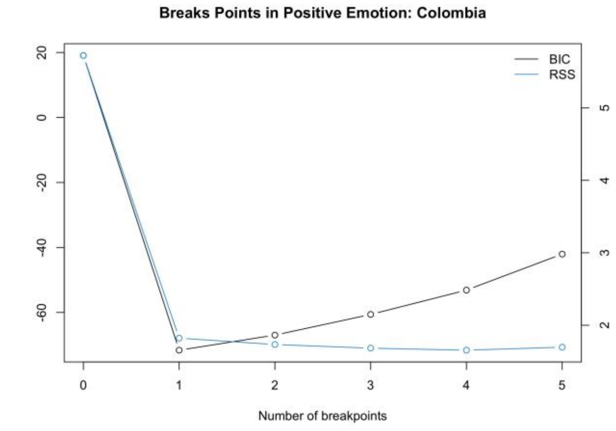

(B) Distribution of F statistics for SupF test.

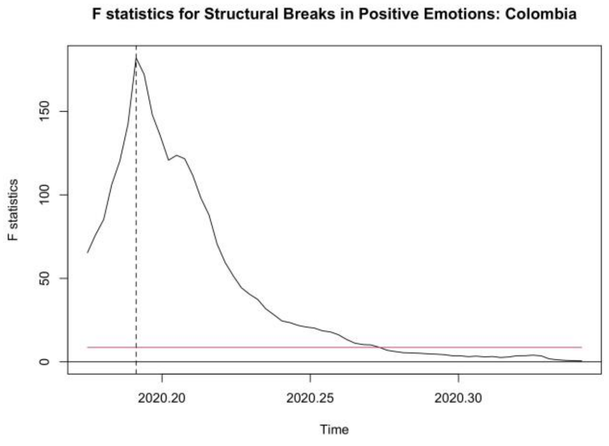

(C) BIC suggested Breaks and confidence interval

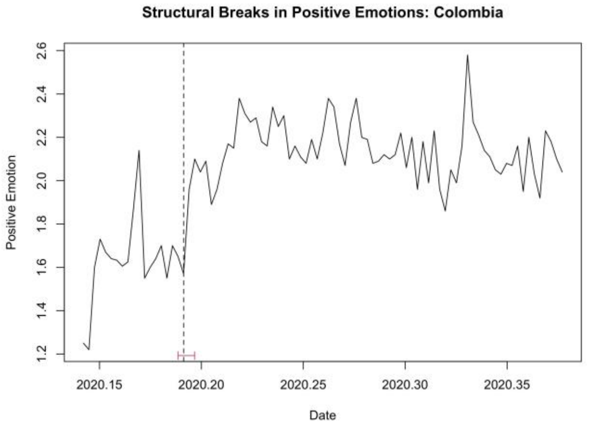

BIC suggested one break on 11/03/2020 (CI = 3)

(D) Plot of Structural Break with segment averages for finally retained breaks.

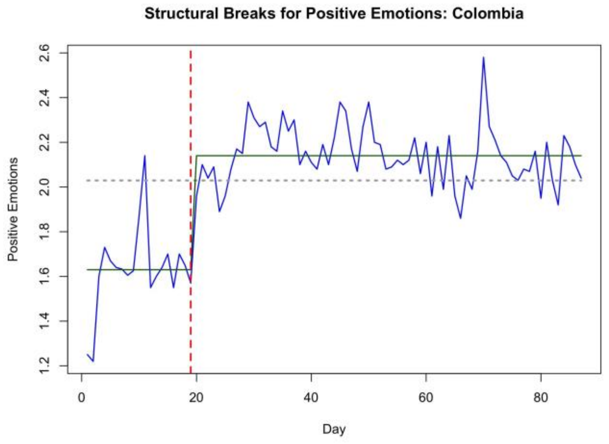

Breaks date = 11/03/2020

Segment averages: Segment1 = 1.63; Segment 2 = 2.14

Figure 12: Plots for Anxiety - Colombia

(A) The BIC and RSS values over number of breakpoints

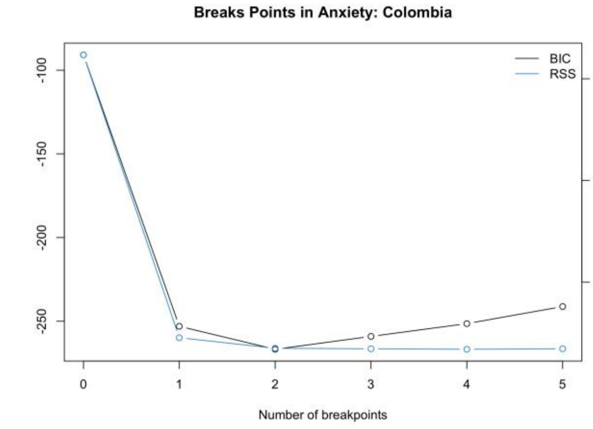

(B) Distribution of F statistics for SupF test.

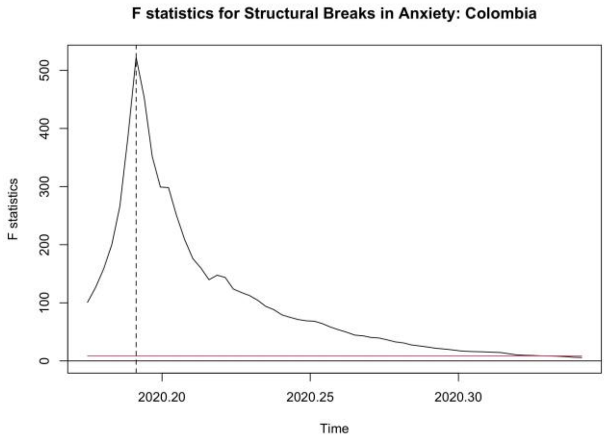

(C) BIC suggested Breaks with Confidence Interval

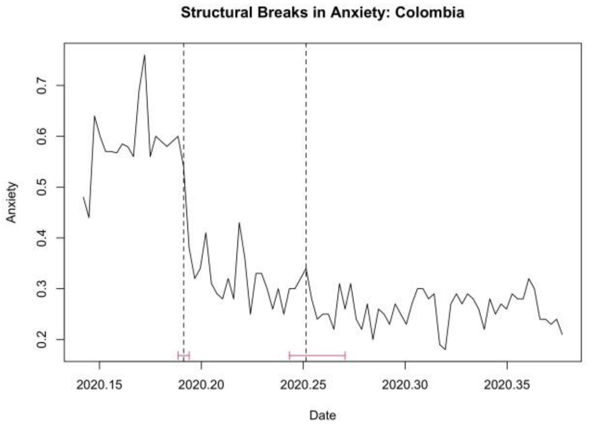

BIC suggested two breaks on 11/03/2020 (CI = 2) and 02/04/2020 (CI = 10)

(D) Plot of Structural Break with segment averages for finally retained breaks.

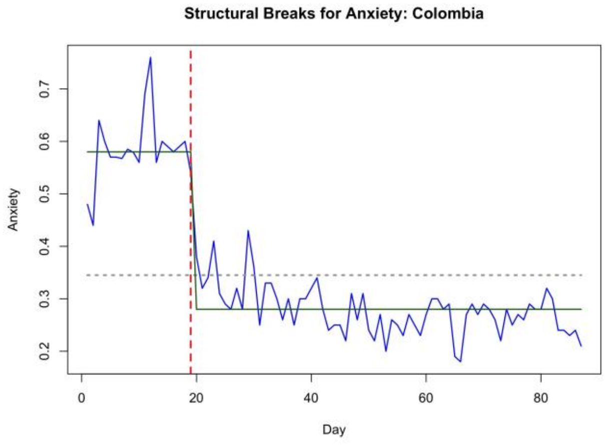

Breaks date = 11/03/2020

Segment averages: Segment1 = 0.58; Segment 2 = 0.28.

France

Figure 13: Plots for Positive Emotion – France

(A) The BIC and RSS values over number of breakpoints

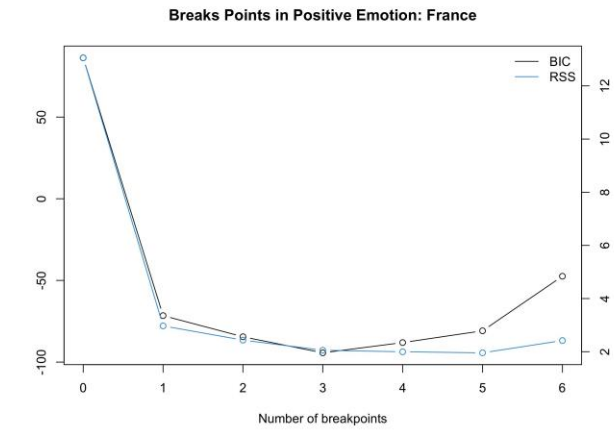

(B) Distribution of F statistics for SupF test.

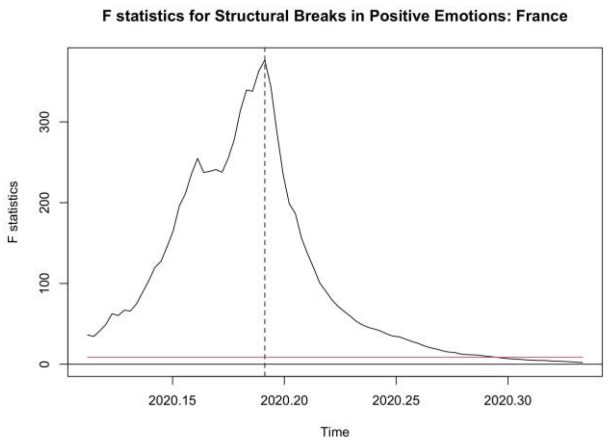

(C) BIC suggested Breaks and confidence interval

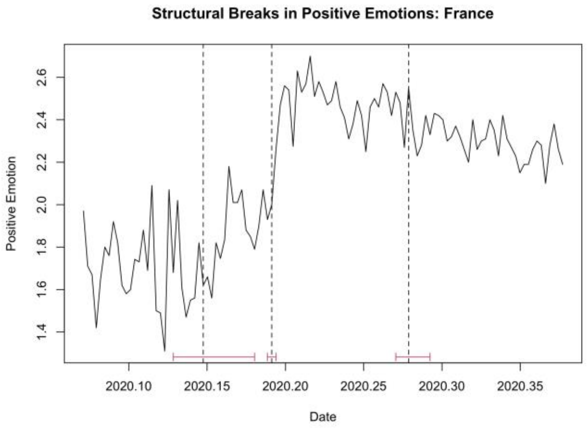

BIC suggested three breaks on 24/02/2020 (CI = 19), and 11/03/2020 (CI = 2), and 12/04/2020 (CI = 8).

(D) Plot of Structural Break with segment averages for finally retained breaks.

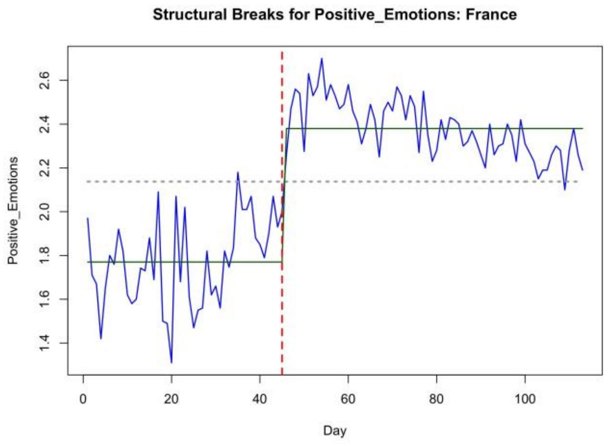

Breaks date = 11/03/2020

Segment averages: Segment1 = 1.77; Segment 2 = 2.38

**Figure 14: Plots for Anxiety - France**	
**(A) The BIC and RSS values over number of breakpoints**<br>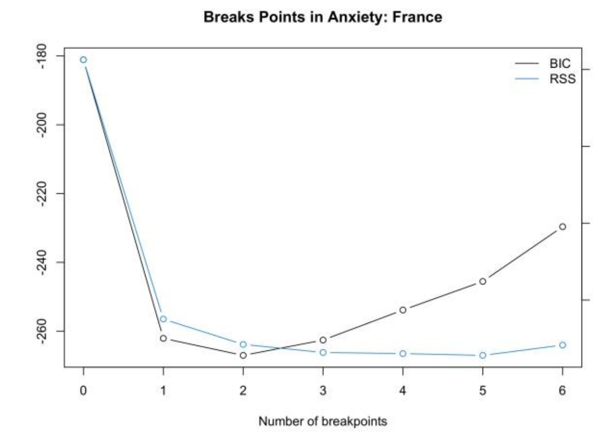	**(B) Distribution of F statistics for SupF test.**<br>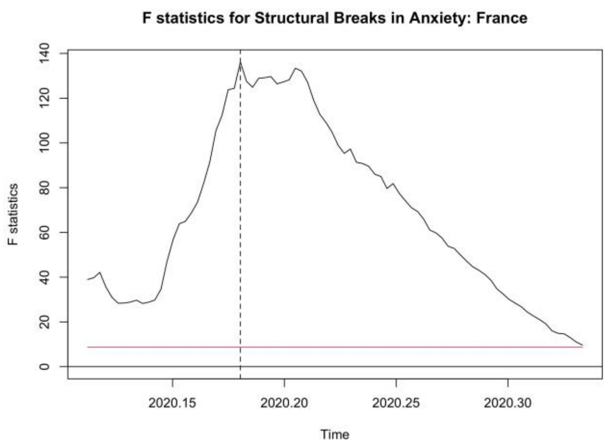
**(C) BIC suggested Breaks with Confidence Interval**<br>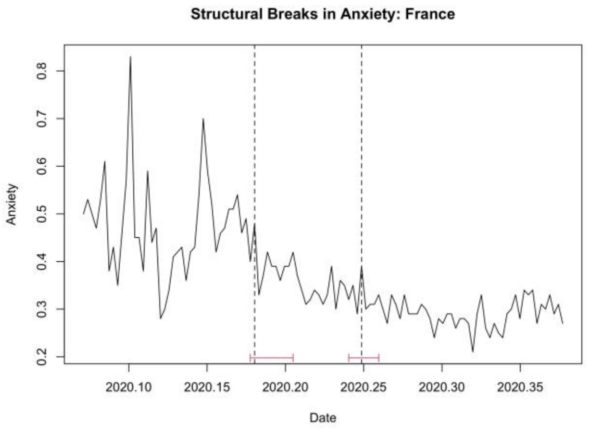<br>BIC suggested two breaks on 07/03/2020 (CI = 10) and 01/04/2020 (CI = 7)	**(D) Plot of Structural Break with segment averages for finally retained breaks.**<br>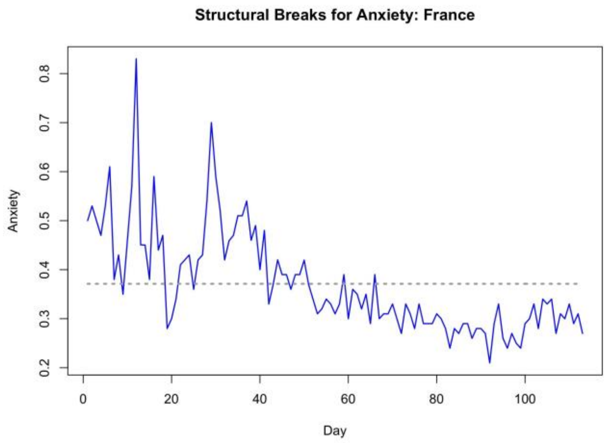<br>Breaks date = None<br>**Series average**: Series Average = 0.37;

Germany

Figure 15: Plots for Positive Emotion – Germany

(A) The BIC and RSS values over number of breakpoints

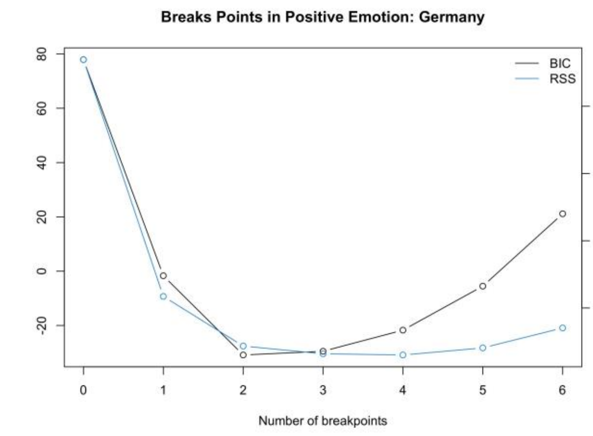

(B) Distribution of F statistics for SupF test.

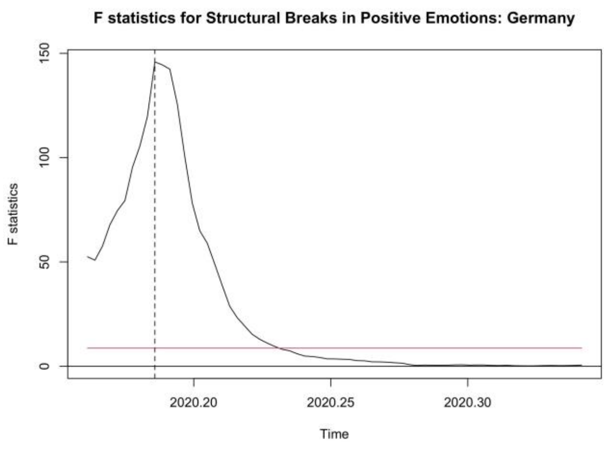

(C) BIC suggested Breaks and confidence interval

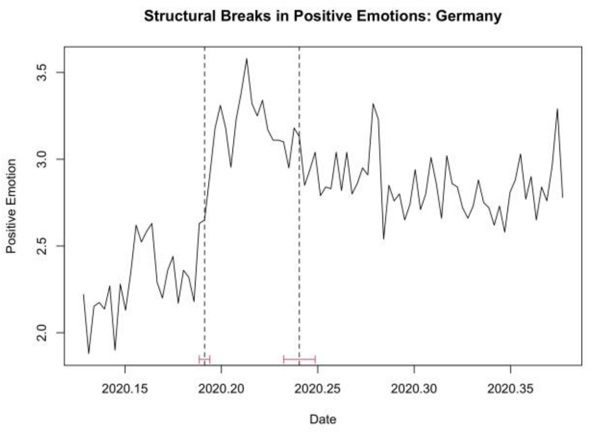

BIC suggested two breaks on 11/03/2020 (CI = 2) and 29/03/2020 (CI = 6)

(D) Plot of Structural Break with segment averages for finally retained breaks.

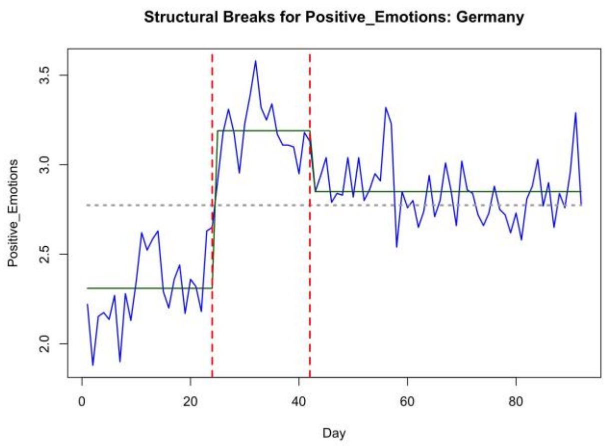

Breaks date = 11/03/2020 and 29/03/2020

Segment averages: Segment1 = 2.31; Segment 2 = 3.19; Segment 3 = 2.85

Figure 16: Plots for Anxiety - Germany

(A) The BIC and RSS values over number of breakpoints

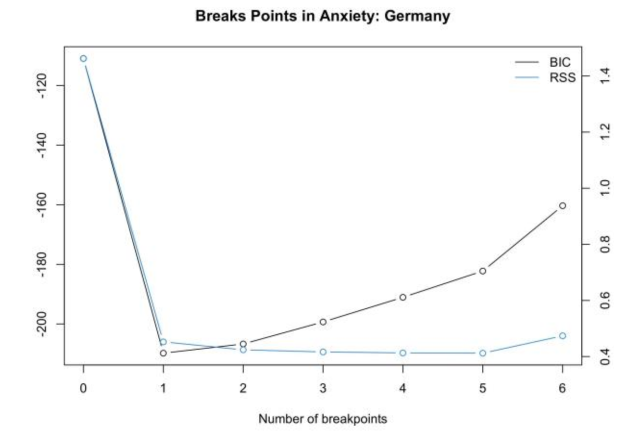

(B) Distribution of F statistics for SupF test.

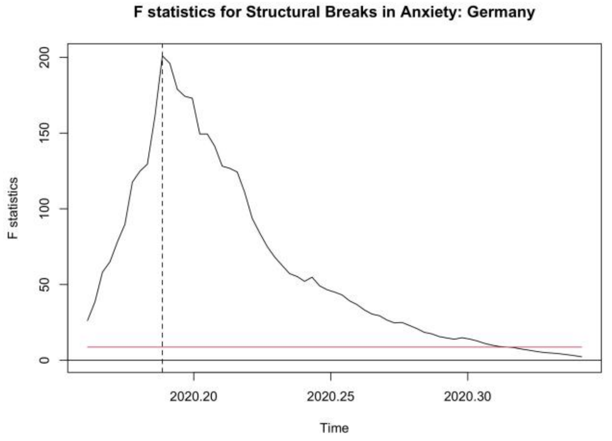

(C) BIC suggested Breaks with Confidence Interval

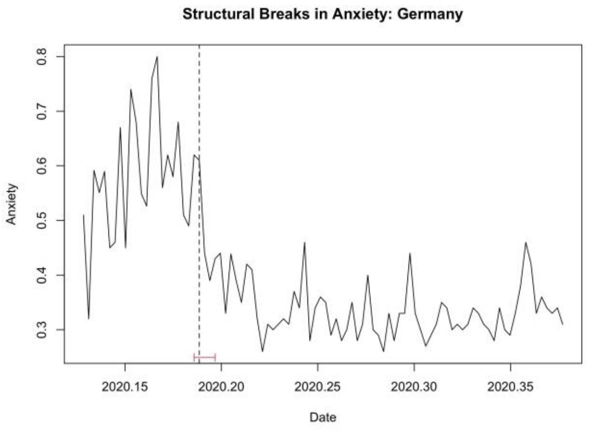

BIC suggested one break on 10/03/2020 (CI = 4)

(D) Plot of Structural Break with segment averages for finally retained breaks

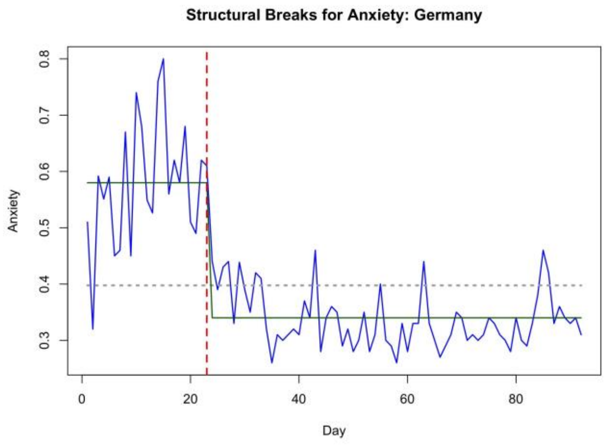

Breaks date = 10/03/2020

Segment averages: Segment1 = 0.58; Segment 2 = 0.34.

India

Figure 17: Plots for Positive Emotion – India

(A) The BIC and RSS values over number of breakpoints

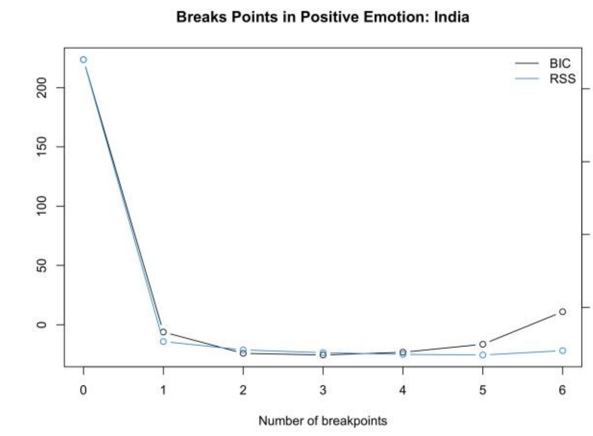

(B) Distribution of F statistics for SupF test.

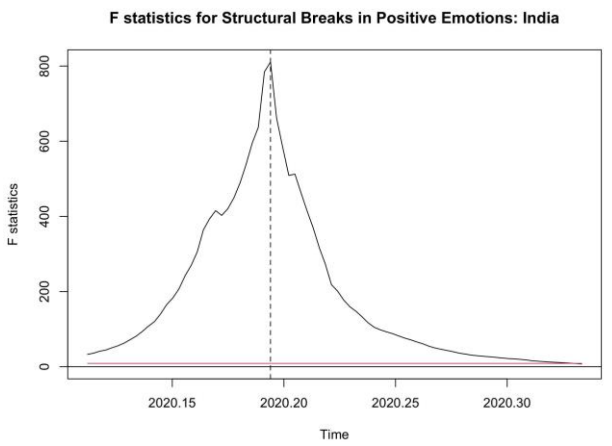

(C) BIC suggested Breaks and confidence interval

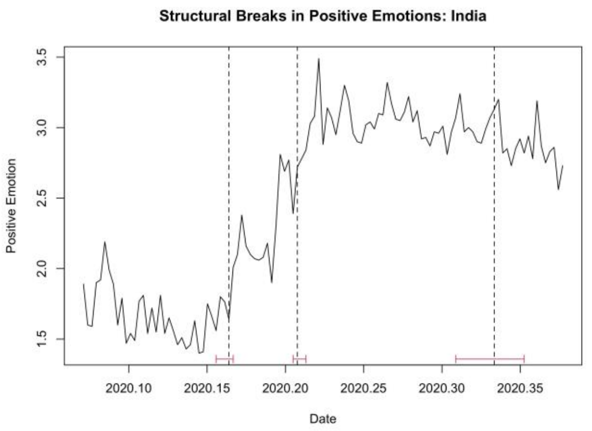

BIC suggested three breaks on 01/03/2020 (CI = 4) and 17/03/2020 (CI = 3) and 02/05/2020 (CI = 16)

(D) Plot of Structural Break with segment averages for finally retained breaks.

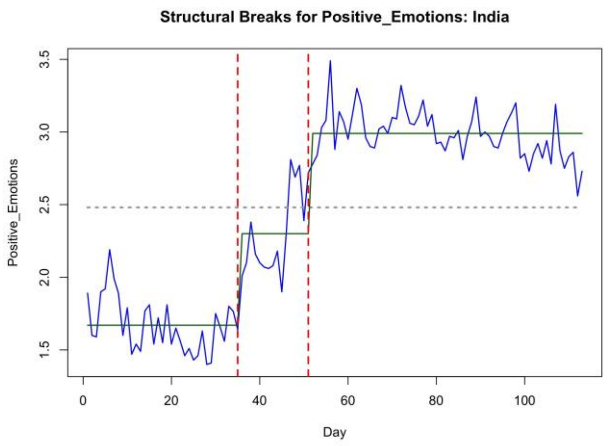

Breaks date = 01/03/2020 and 17/03/2020

Segment averages: Segment1 = 1.67; Segment 2 = 2.30; Segment 2 = 2.99

Figure 18: Plots for Anxiety - India

(A) The BIC and RSS values over number of breakpoints	(B) Distribution of F statistics for SupF test.
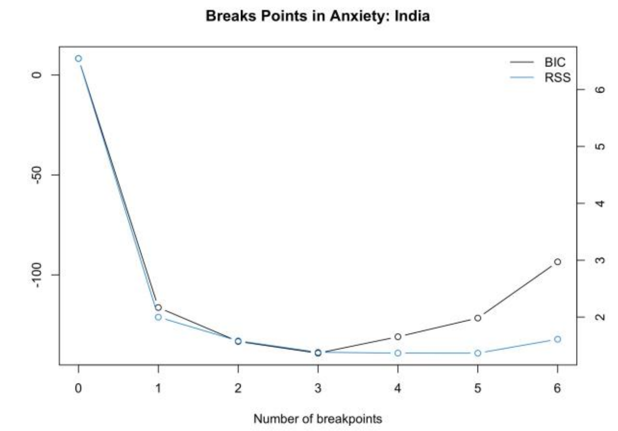	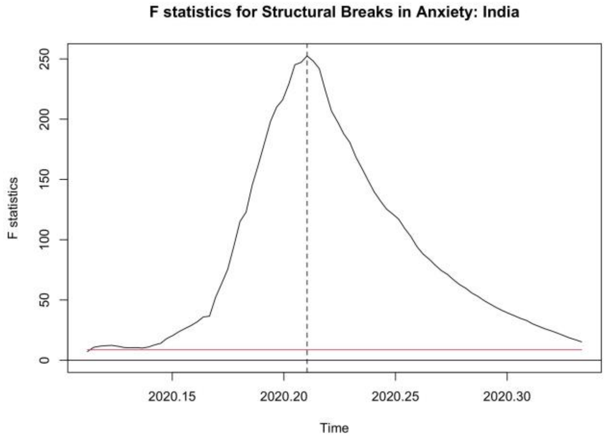
**(C) BIC suggested Breaks with Confidence Interval**	**(D) Plot of Structural Break with segment averages for finally retained breaks.**
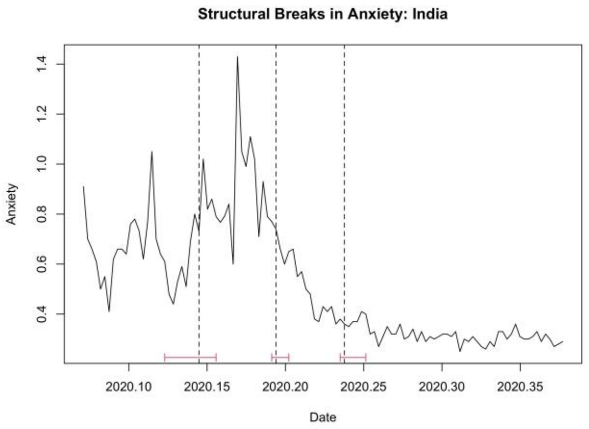	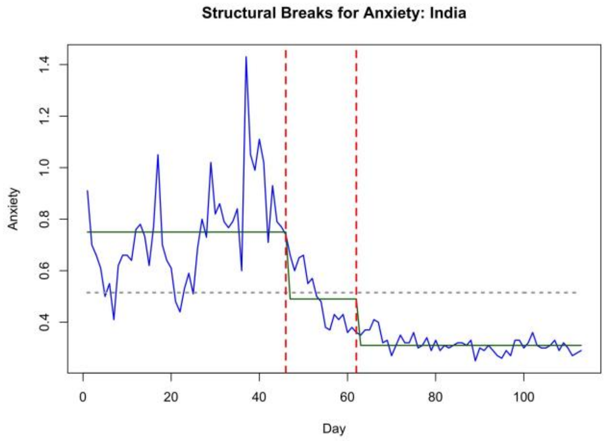
BIC suggested three breaks on 23/02/2020 (CI = 12) and 12/03/2020 (CI = 4) and 28/03/2020 (CI = 6)	Breaks date = 12/03/2020 and 28/03/2020<br>**Segment averages**: Segment1 = 0.75; Segment 2 = 0.49; Segment 3 = 0.31.

Mexico

Figure 19: Plots for Positive Emotion – Mexico

(A) The BIC and RSS values over number of breakpoints

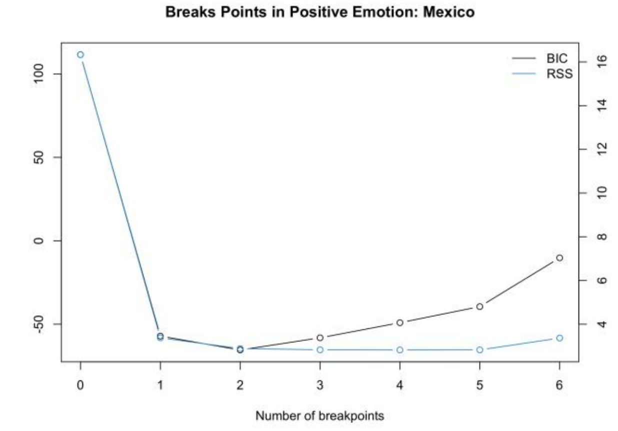

(B) Distribution of F statistics for SupF test.

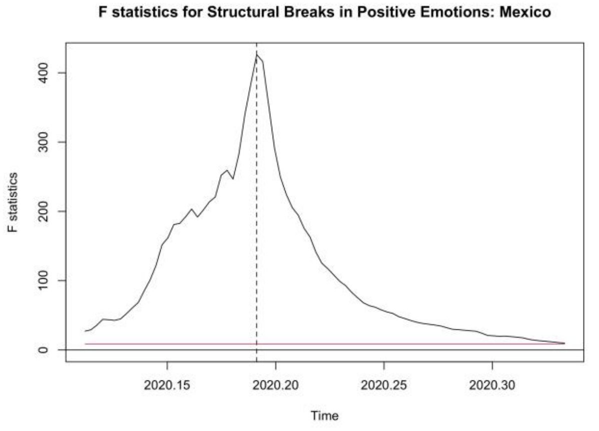

(C) BIC suggested Breaks and confidence interval

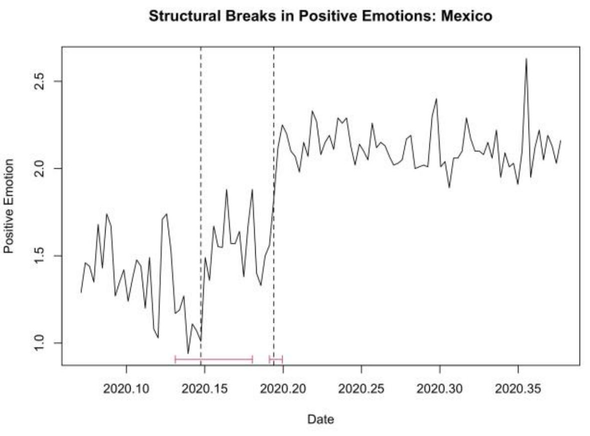

BIC suggested two breaks on 24/02/2020 (CI = 18) and 12/02/2020 (CI = 3)

(D) Plot of Structural Break with segment averages for finally retained breaks.

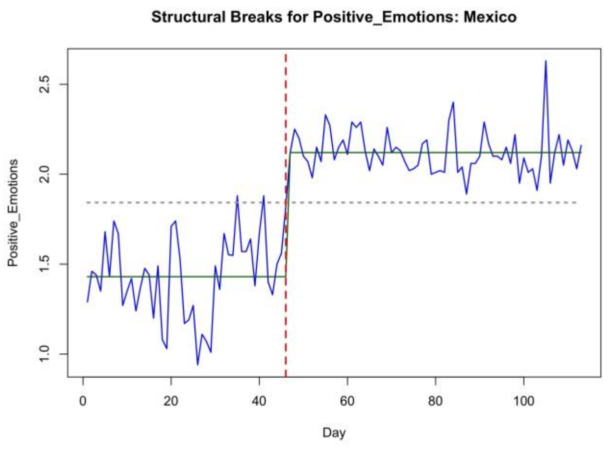

Breaks date = 12/03/2020

Segment averages: Segment1 = 1.43; Segment 2 = 2.12

Figure 20: Plots for Anxiety – Mexico

**(A) The BIC and RSS values over number of breakpoints**	**(B) Distribution of F statistics for SupF test.**
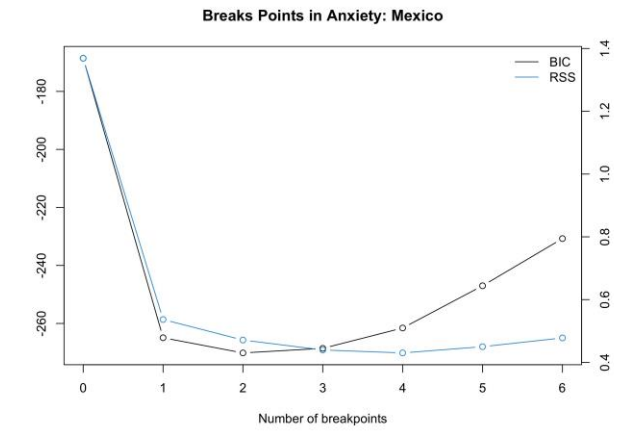	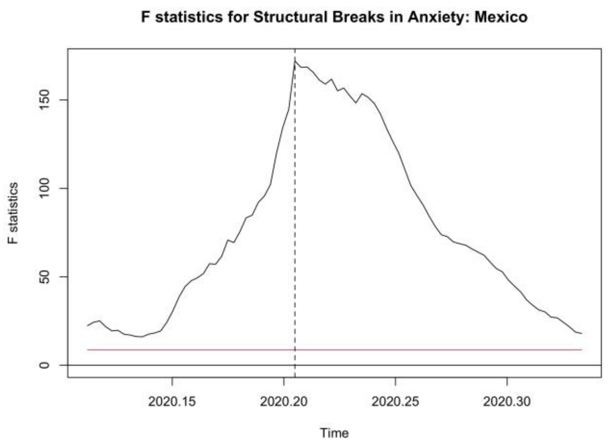
**(C) BIC suggested Breaks with Confidence Interval**	**(D) Plot of Structural Break with segment averages for finally retained breaks**
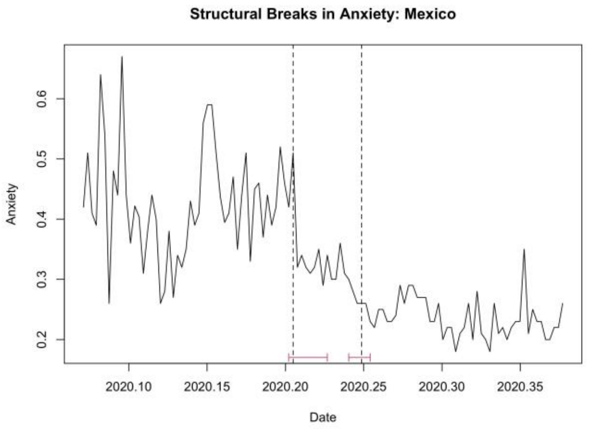 BIC suggested two breaks on 16/03/2020 (CI = 9) and 01/04/2020 (CI = 5)	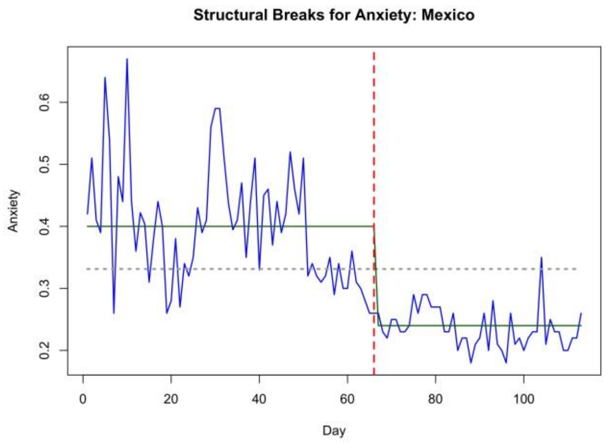 Breaks date = 01/04/2020<br><br>**Segment averages**: Segment1 = 0.40; Segment 2 = 0.24.

New Zealand

Figure 21: Plots for Positive Emotion – New Zealand

(A) The BIC and RSS values over number of breakpoints

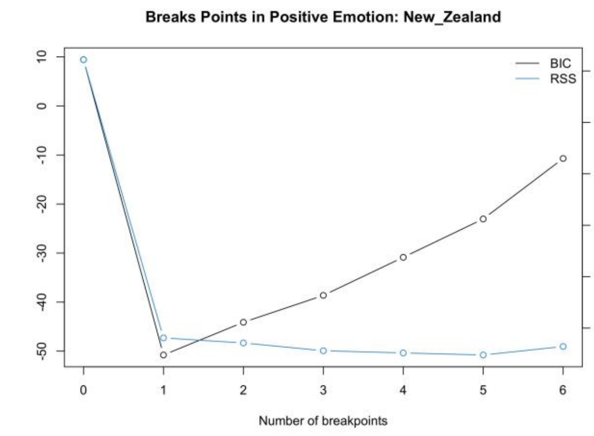

(B) Distribution of F statistics for SupF test.

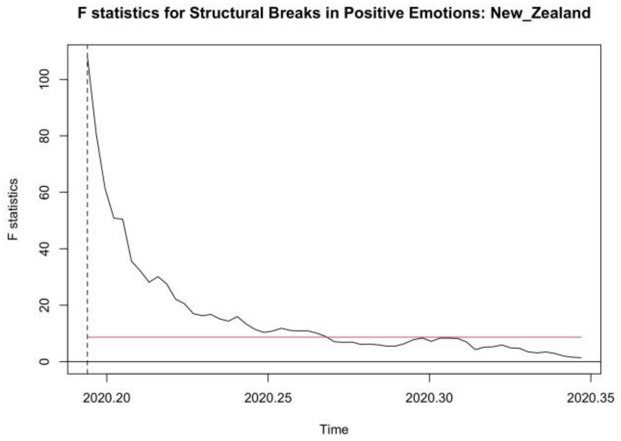

(C) BIC suggested Breaks and confidence interval

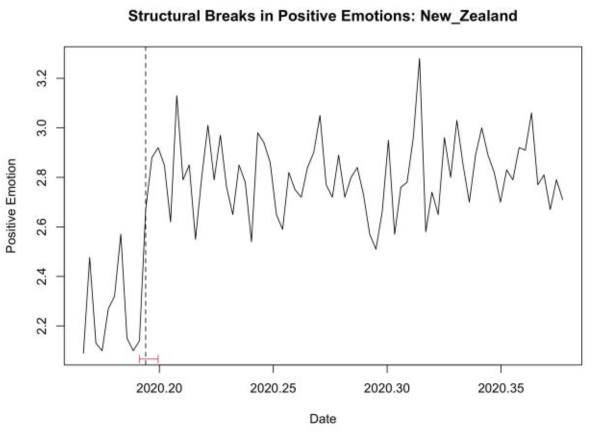

BIC suggested one break on 12/03/2020 (CI = 3)

(D) Plot of Structural Break with segment averages for finally retained breaks.

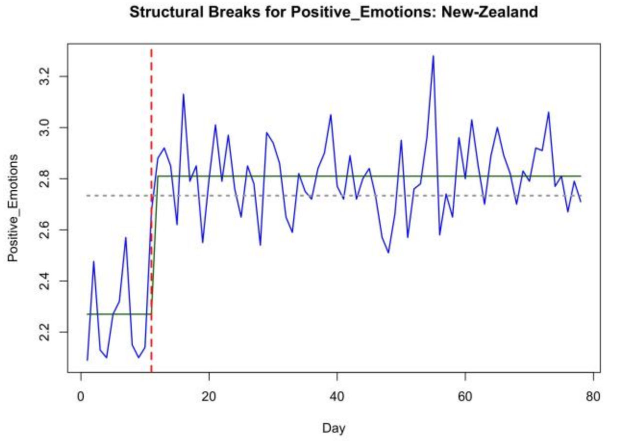

Breaks date = 12/03/2020

Segment averages: Segment1 = 2.27; Segment 2 = 2.81

Figure 22: Plots for Anxiety - New Zealand

(A) The BIC and RSS values over number of breakpoints	(B) Distribution of F statistics for SupF test.
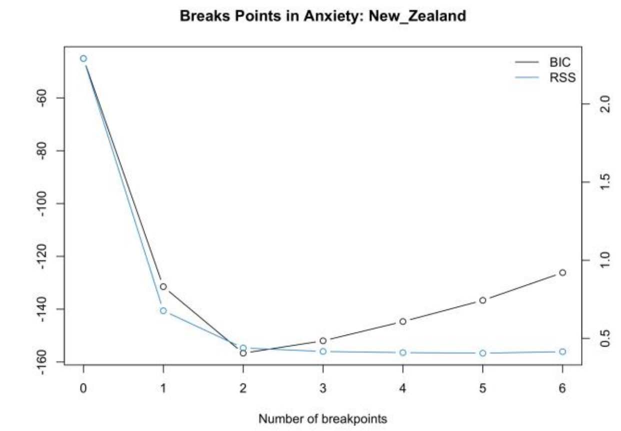	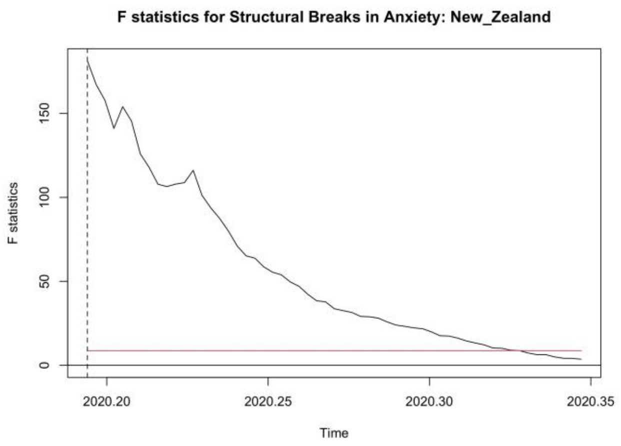
**(C) BIC suggested Breaks with Confidence Interval**	**(D) Plot of Structural Break with segment averages for finally retained breaks.**
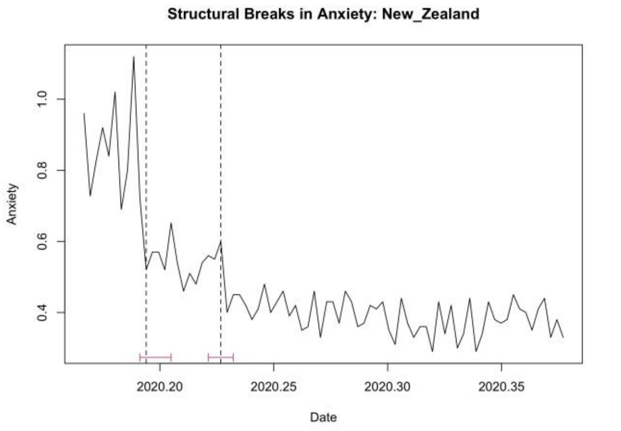<br>BIC suggested two breaks on 12/03/2020 (CI = 5) and 24/03/2020 (CI = 4)	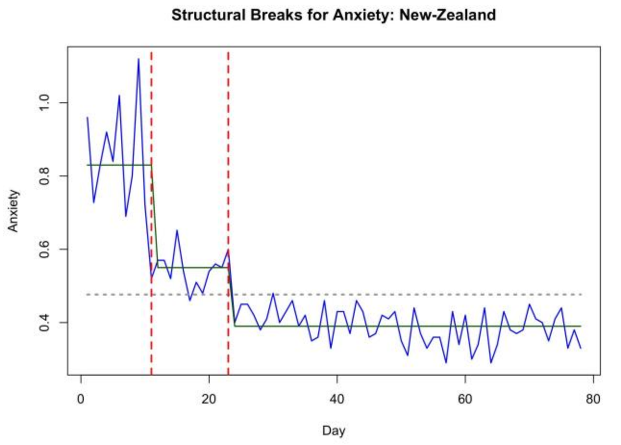<br>Breaks date = 12/03/2020 and 24/03/2020<br>**Segment averages**: Segment1 = 0.83; Segment 2 = 0.55; Segment 2 = 0.39.

Nigeria

**Figure 23: Plots for Positive Emotion – Nigeria**	
**(A) The BIC and RSS values over number of breakpoints**<br>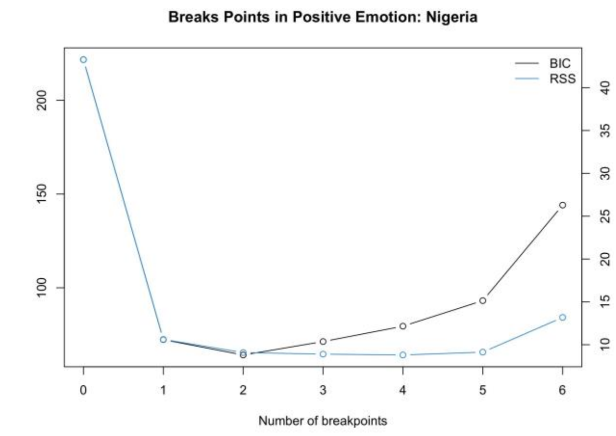 	**(B) Distribution of F statistics for SupF test.**<br>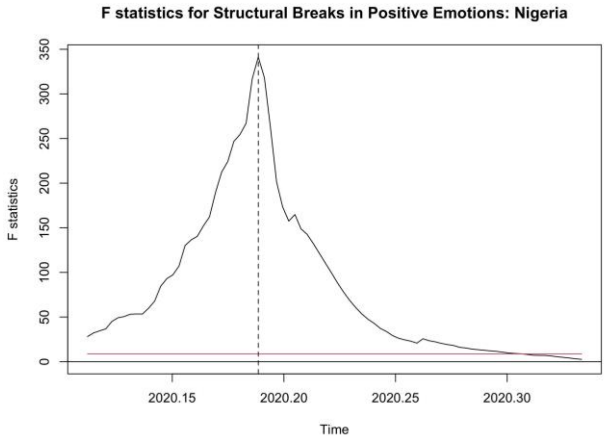
**(C) BIC suggested Breaks and confidence interval**<br>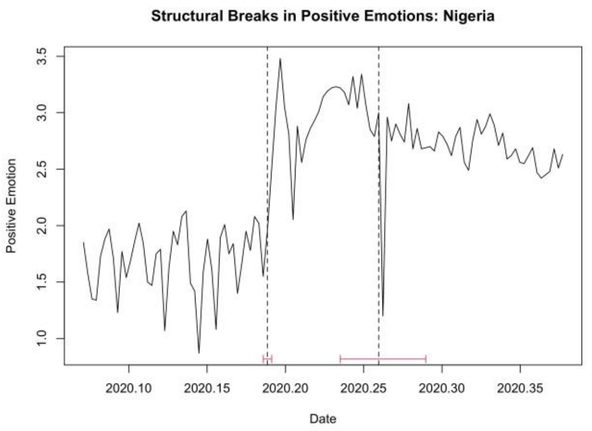<br><br>BIC suggested two breaks on 10/03/2020 (CI = 2) and 05/04/2020 (CI = 20)	**(D) Plot of Structural Break with segment averages for finally retained breaks**<br>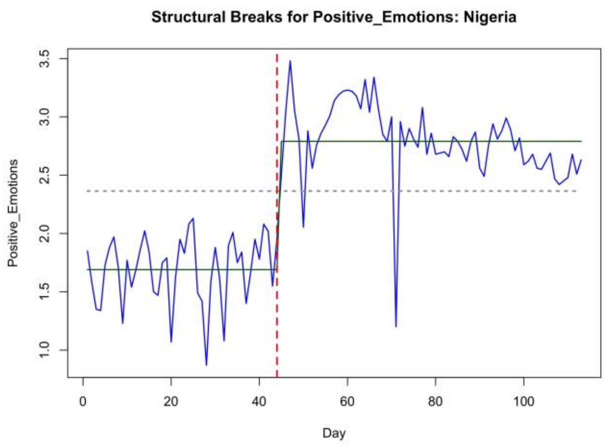<br><br>Breaks date = 10/03/2020<br>**Segment averages**: Segment1 = 1.69; Segment 2 = 2.79

**Figure 24: Plots for Anxiety - Nigeria**

<table>
<tr>
<td>(A) The BIC and RSS values over number of breakpoints
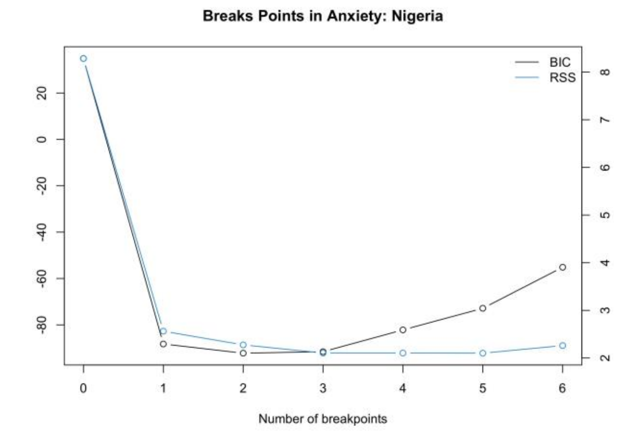</td>
<td>(B) Distribution of F statistics for SupF test.
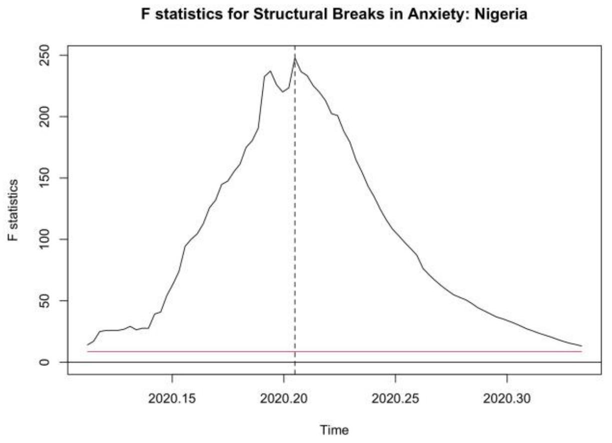</td>
</tr>
<tr>
<td>(C) BIC suggested Breaks with Confidence Interval
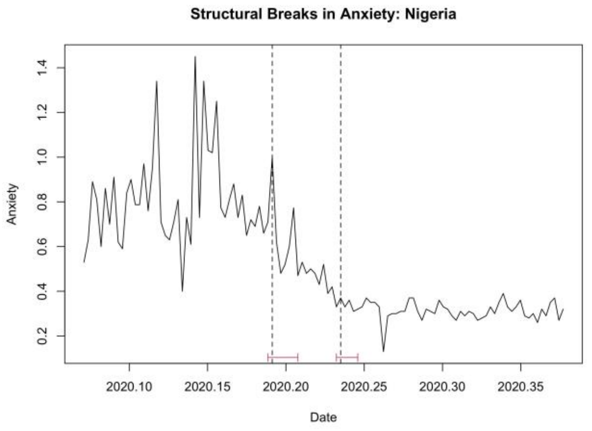
BIC suggested two breaks on 11/03/2020 (CI = 7) and 27/03/2020 (CI = 5)</td>
<td>(D) Plot of Structural Break with segment averages for finally retained breaks
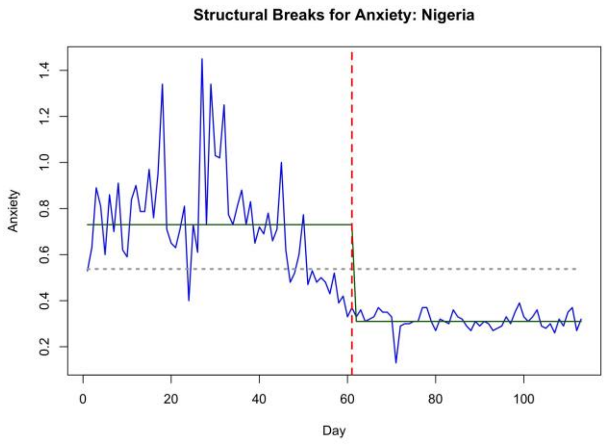
Breaks date = 27/03/2020
Segment averages: Segment1 = 0.73; Segment 2 = 0.31.</td>
</tr>
</table>

Singapore:

Figure 27: Plots for Positive Emotion – Singapore

(A) The BIC and RSS values over number of breakpoints

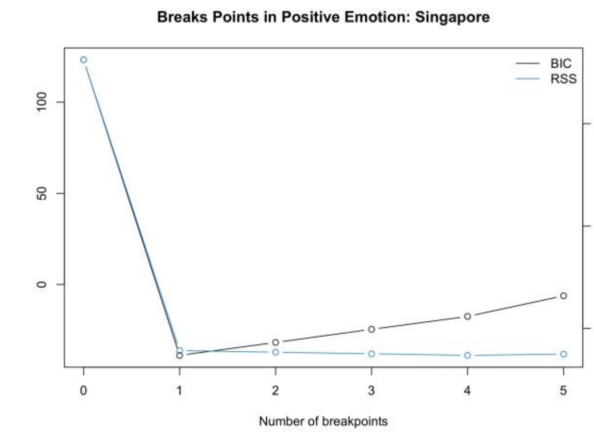

(B) Distribution of F statistics for SupF test.

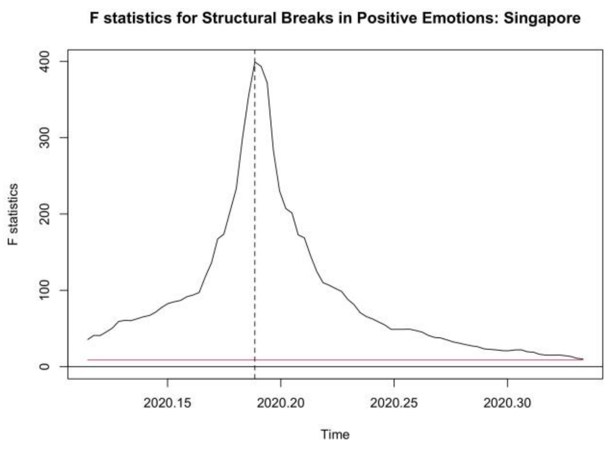

(C) BIC suggested Breaks with Confidence Interval

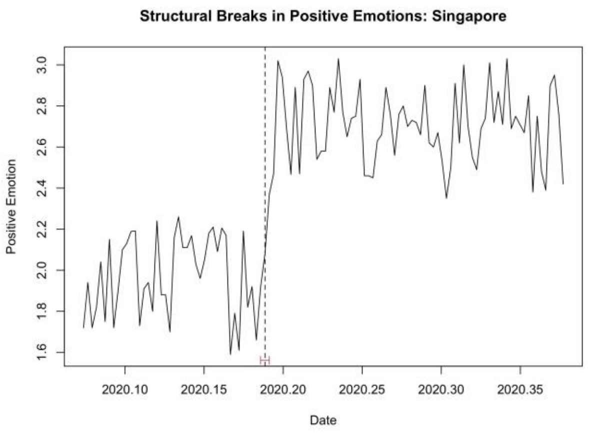

BIC suggested one break on 10/03/2020 (CI = 2)

(D) Plot of Structural Break with segment averages for finally retained breaks.

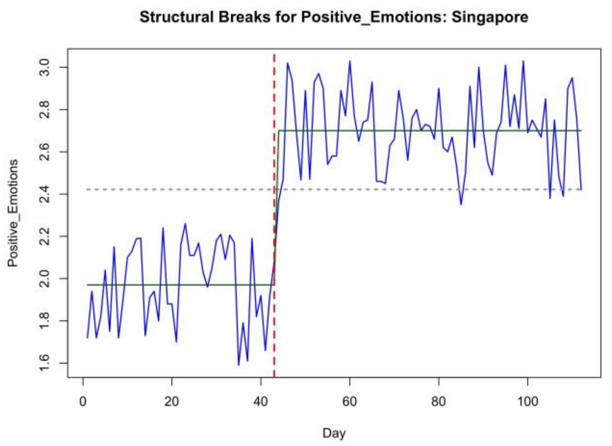

Breaks date = 10/03/2020

Segment averages: Segment1 = 1.97; Segment 2 = 2.70

<table>
<tr><td colspan="2">Figure 28: Plots for Anxiety – Singapore</td></tr>
<tr><td>

(A) The BIC and RSS values over number of breakpoints

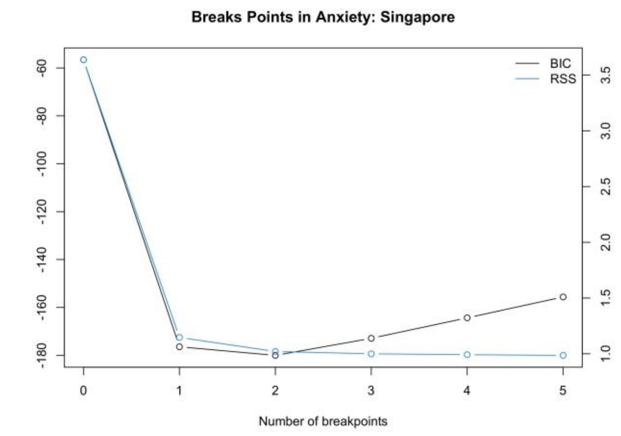

</td><td>

(B) Distribution of F statistics for SupF test.

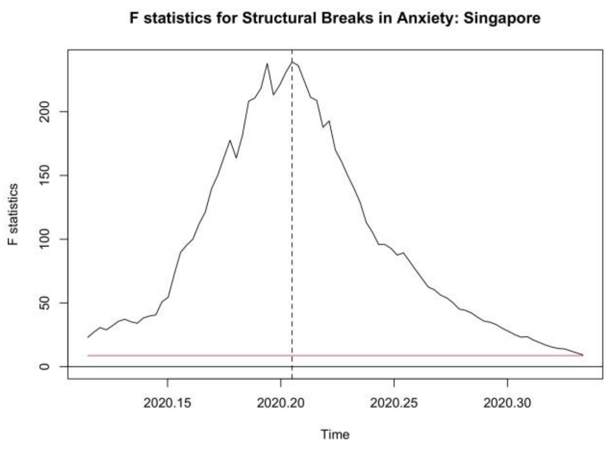

</td></tr>
<tr><td>

(C) BIC suggested Breaks with Confidence Interval

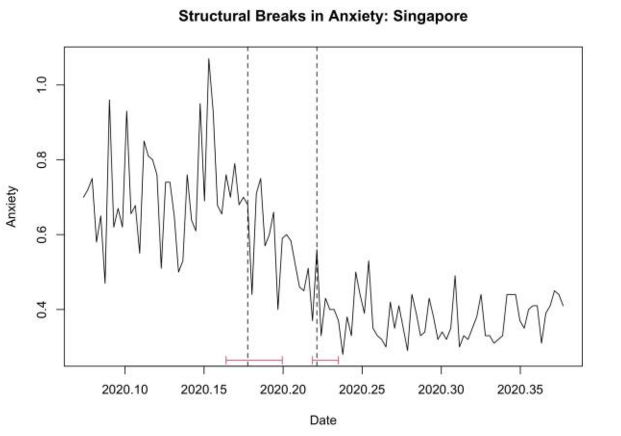

BIC suggested two breaks on 06/03/2020 (CI = 13) and 22/03/2020 (CI = 6)

</td><td>

(D) Plot of Structural Break with segment averages for finally retained breaks.

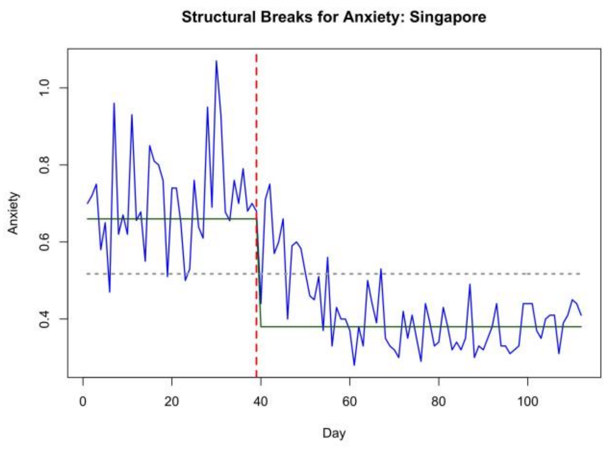

Breaks date = 22/03/2020

Segment averages: Segment1 = 0.66; Segment 2 = 0.38

</td></tr>
</table>

South Africa:

Figure 29: Plots for Positive Emotion - South Africa

(A) The BIC and RSS values over number of breakpoints

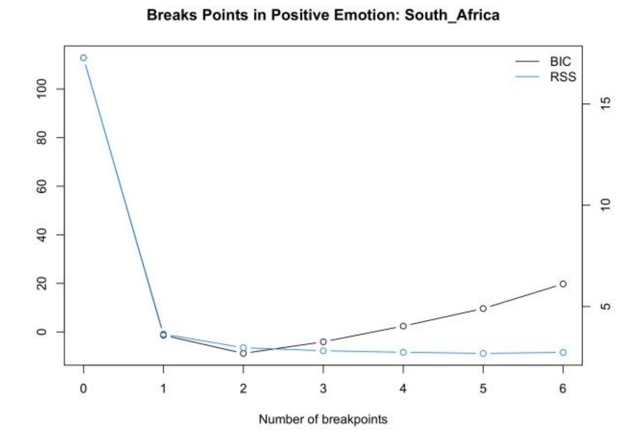

(B) Distribution of F statistics for SupF test.

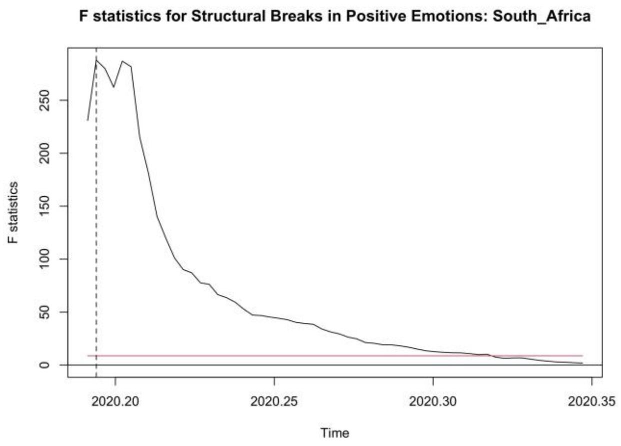

(C) BIC suggested Breaks with Confidence Interval

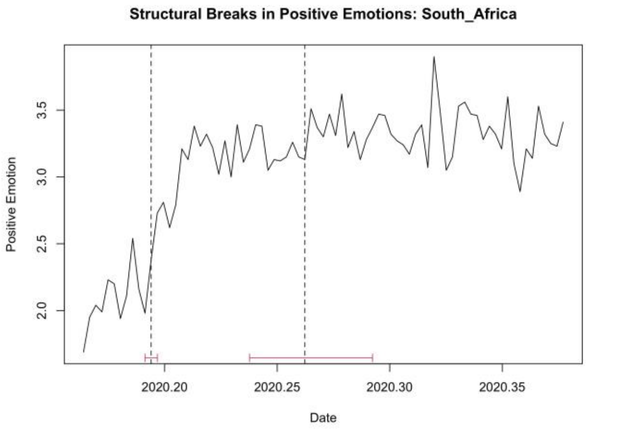

BIC suggested two breaks on 12/03/2020 (CI = 02) and 06/04/2020 (CI = 20)

(D) Plot of Structural Break with segment averages for finally retained breaks

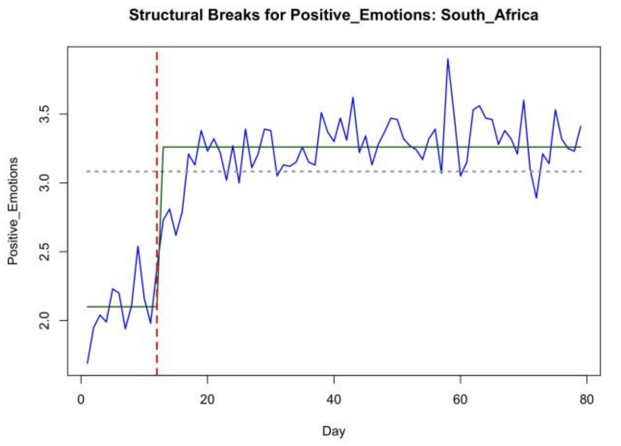

Breaks date = 12/03/2020

Segment averages: Segment1 = 2.10; Segment 2 = 3.26

<table>
<tr><td colspan="2">Figure 30: Plots for Anxiety - South Africa</td></tr>
<tr><td>(A) The BIC and RSS values over number of breakpoints
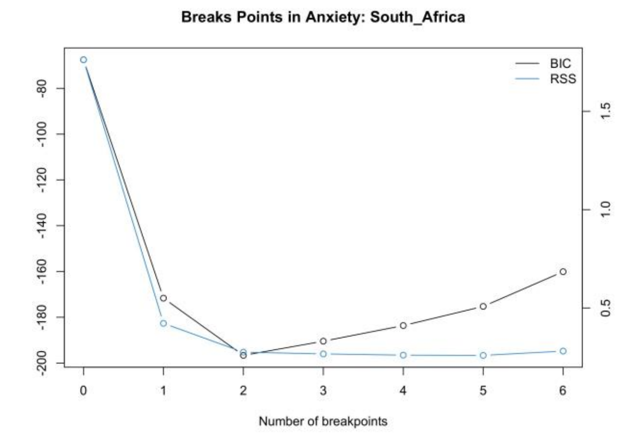
</td>
<td>(B) Distribution of F statistics for SupF test.
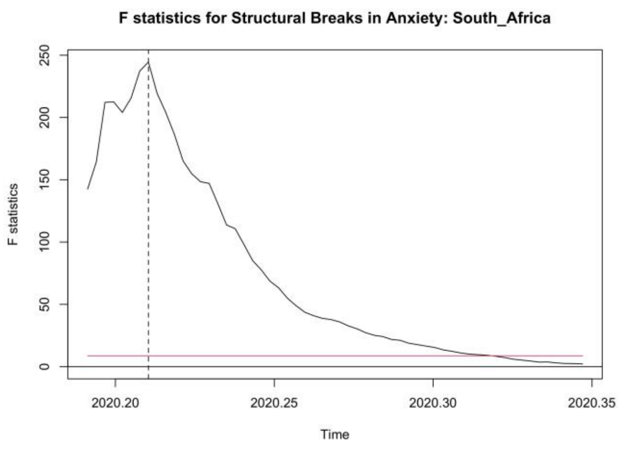
</td></tr>
<tr><td>(C) BIC suggested Breaks with Confidence Interval
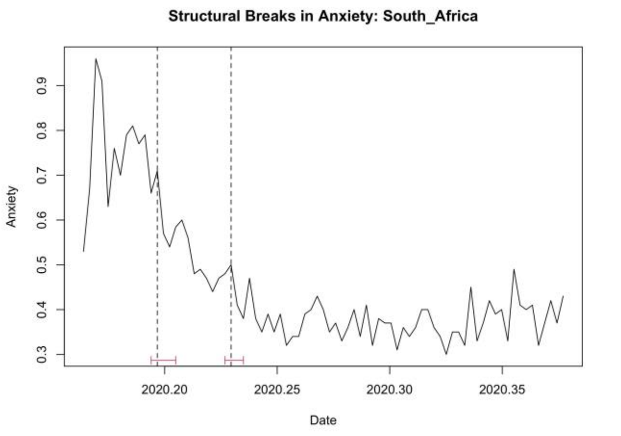

BIC suggested two breaks on 13/03/2020 (CI = 4) and 25/03/2020 (CI = 3)</td>
<td>(D) Plot of Structural Break with segment averages for finally retained breaks
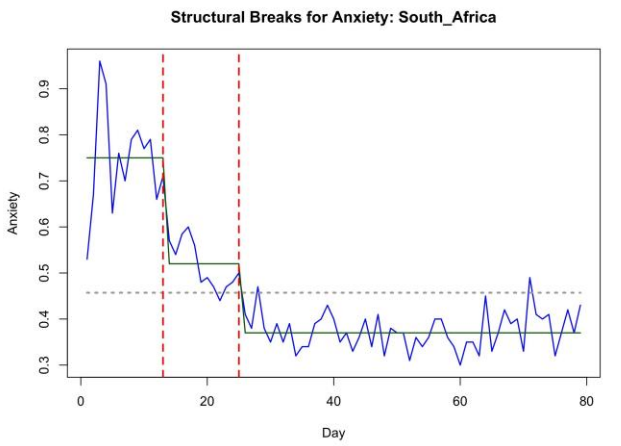

Breaks date = 13/03/2020 and 25/03/2020
Segment averages: Segment1 = 0.75; Segment 2 = 0.52; Segment 3 = 0.37</td></tr>
</table>

Spain:

Figure 31: Plots for Positive Emotion – Spain

(A) The BIC and RSS values over number of breakpoints

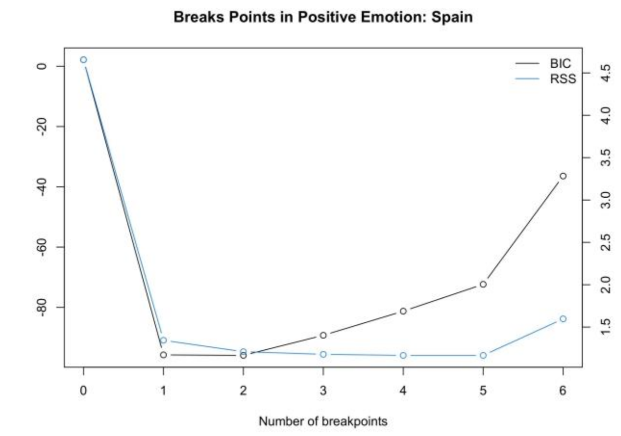

(B) Distribution of F statistics for SupF test.

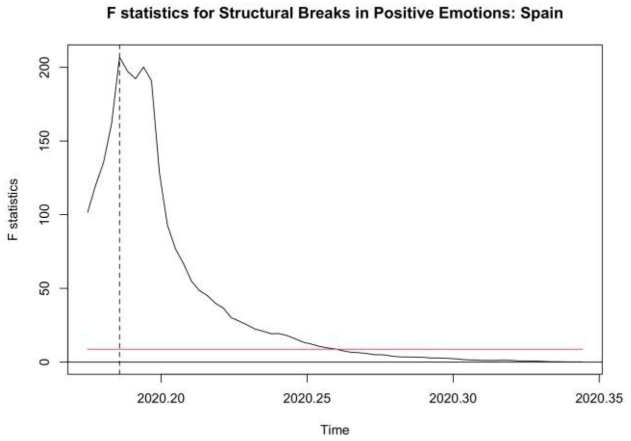

(C) BIC suggested Breaks with Confidence Interval

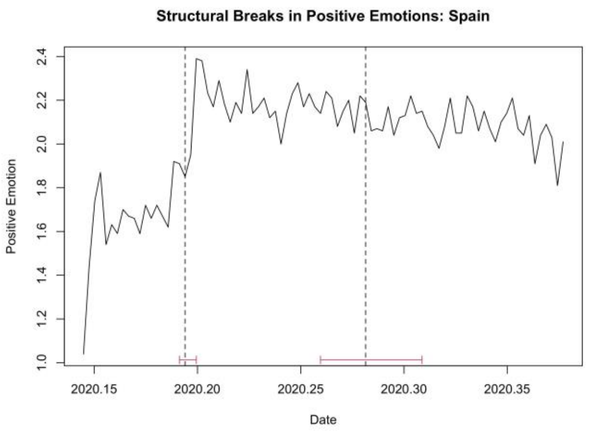

BIC suggested two breaks on 12/03/2020 (CI = 3) and 05/04/2020 (CI = 18)

(D) Plot of Structural Break with segment averages for finally retained breaks

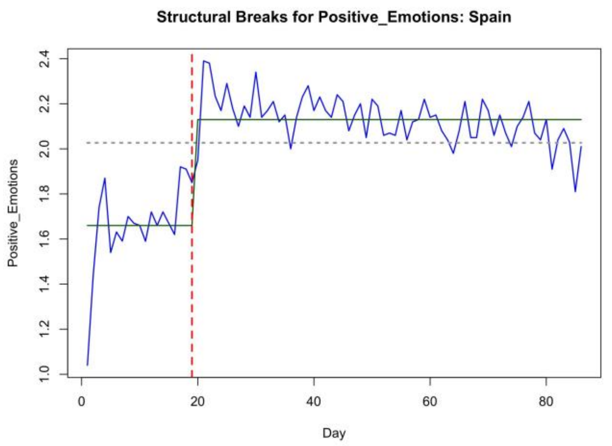

Breaks date = 12/03/2020

Segment averages: Segment1 = 1.66; Segment 2 = 2.13

Figure 32: Plots for Anxiety – Spain

<table>
<tr>
<td>

(A) The BIC and RSS values over number of breakpoints

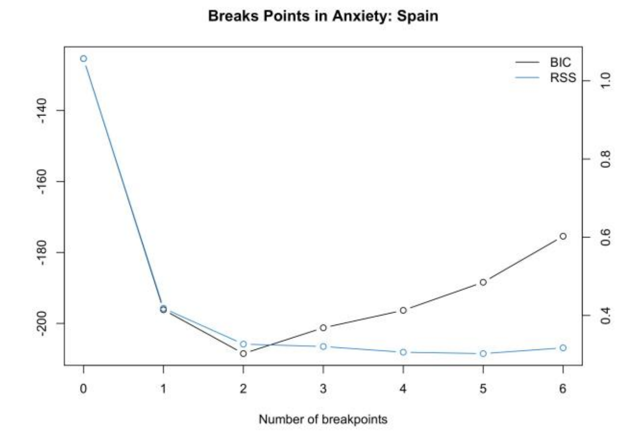

</td>
<td>

(B) Distribution of F statistics for SupF test.

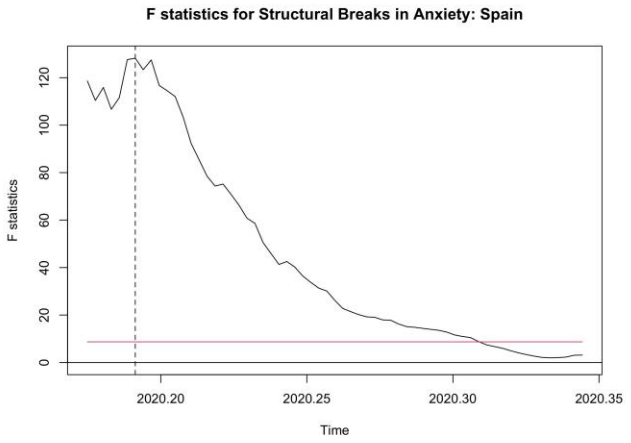

</td>
</tr>
<tr>
<td>

(C) BIC suggested Breaks with Confidence Interval

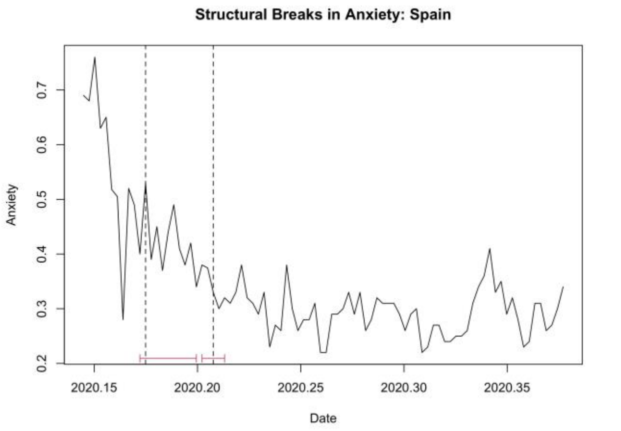

BIC suggested two breaks on 15/03/2020 (CI = 10) and 17/03/2020 (CI = 4)

</td>
<td>

(D) Plot of Structural Break with segment averages for finally retained breaks

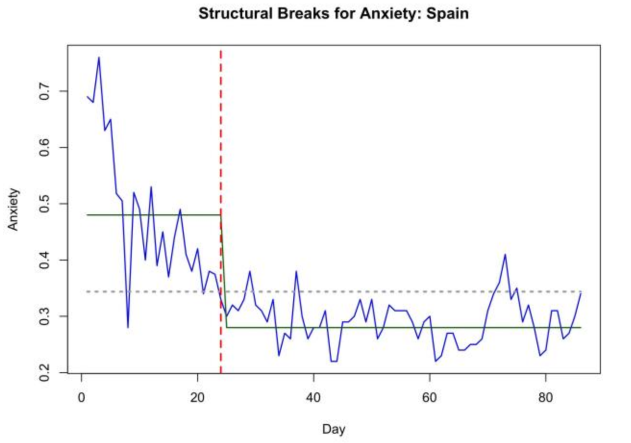

Breaks date = 17/03/2020

Segment averages: Segment1 = 0.48; Segment 2 = 0.29

</td>
</tr>
</table>

United Kingdom:

Figure 33: Plots for Positive Emotion - United Kingdom

(A) The BIC and RSS values over number of breakpoints

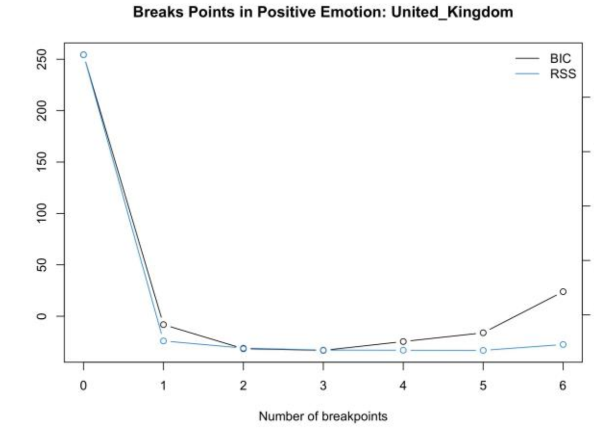

(B) Distribution of F statistics for SupF test.

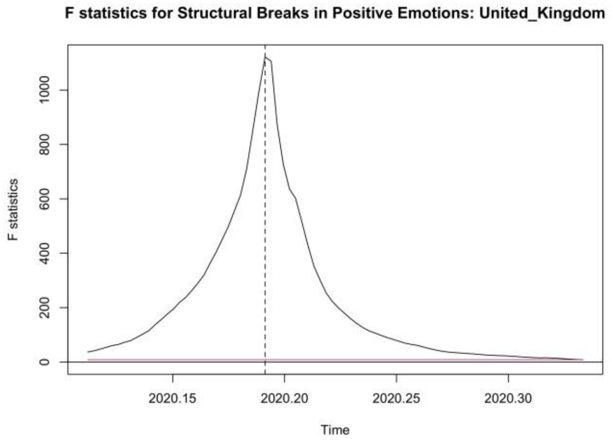

(C) BIC suggested Breaks with Confidence Interval

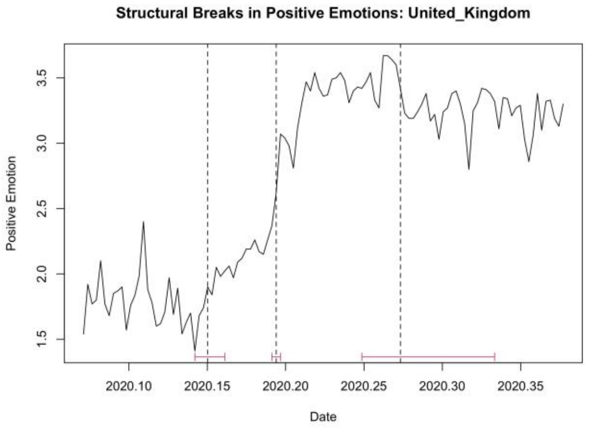

BIC suggested three breaks on 25/02/2020 (CI = 7) and 12/03/2020 (CI = 2) and 10/04/2020 (CI = 31)

(D) Plot of Structural Break with segment averages for finally retained breaks

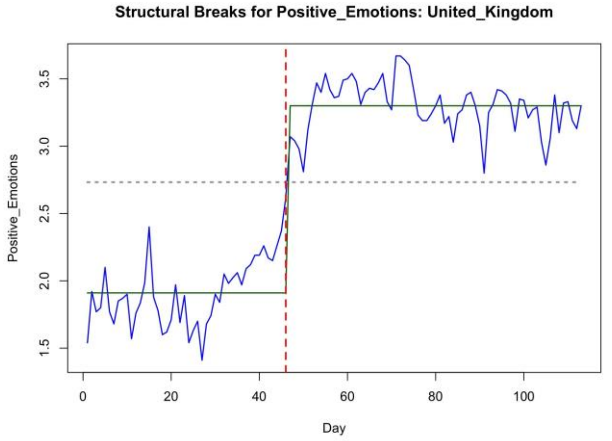

Breaks date = 12/03/2020

Segment averages: Segment1 = 1.91; Segment 2 = 3.30

Figure 34: Plots for Anxiety - United Kingdom

<table>
<tr>
<td>(A) The BIC and RSS values over number of breakpoints
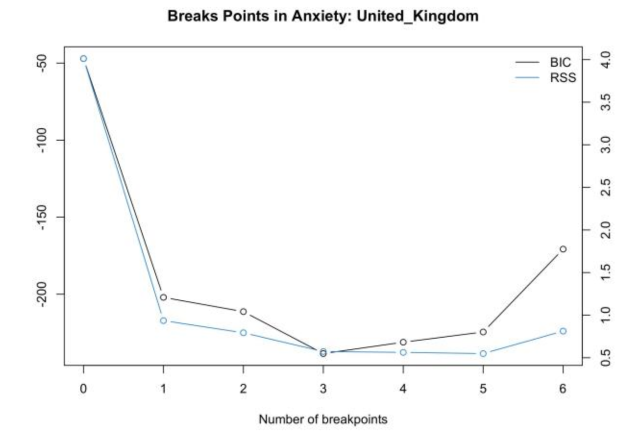
</td>
<td>(B) Distribution of F statistics for SupF test.
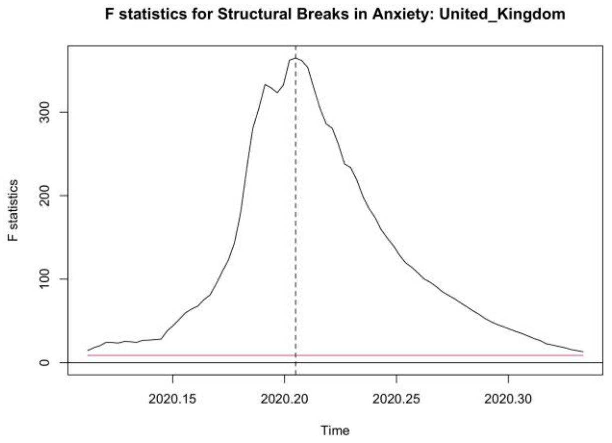
</td>
</tr>
<tr>
<td>(C) BIC suggested Breaks with Confidence Interval
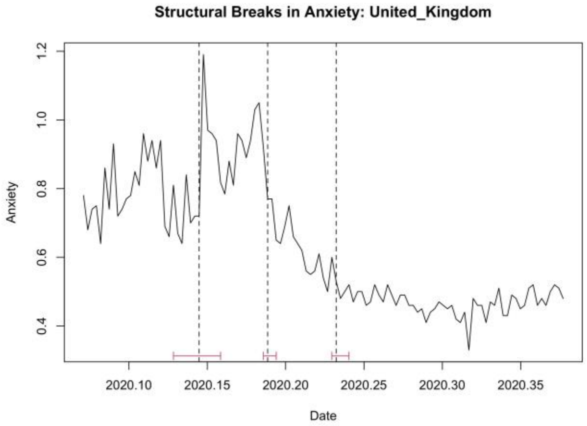

BIC suggested three breaks on 23/02/2020 (CI = 11) and 10/03/2020 (CI = 3) and 26/03/2020 (CI = 4)</td>
<td>(D) Plot of Structural Break with segment averages for finally retained breaks
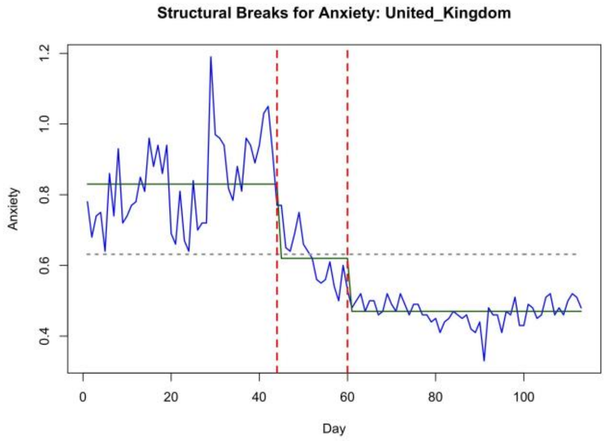

Breaks date = 10/03/2020 and 26/03/2020
Segment averages: Segment1 = 0.83; Segment 2 = 0.62; Segment 3 = 0.47</td>
</tr>
</table>

United States of America:

Figure 35: Plots for Positive Emotion - United States of America

(A) The BIC and RSS values over number of breakpoints

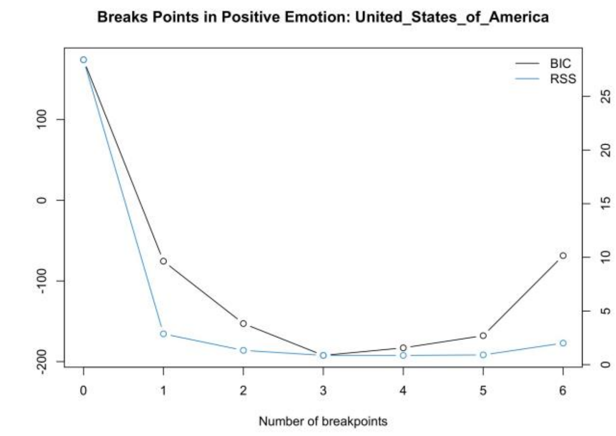

(B) Distribution of F statistics for SupF test.

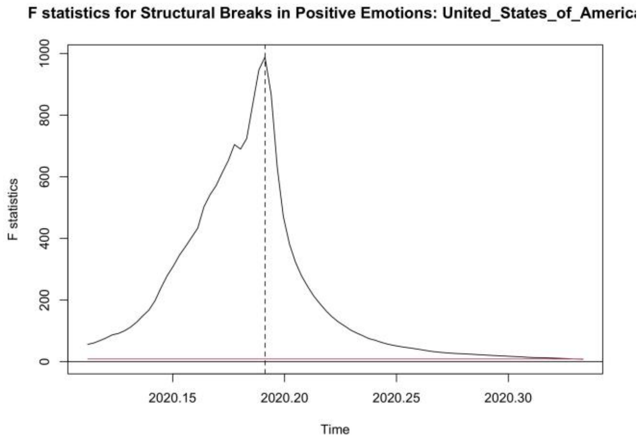

(C) BIC suggested Breaks with Confidence Interval

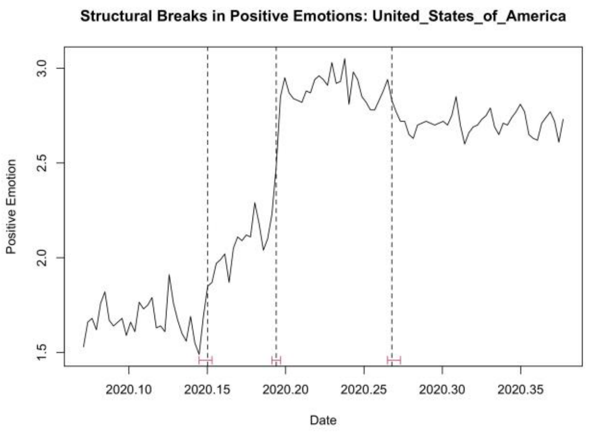

BIC suggested three breaks on 25/02/2020 (CI = 3) and 12/03/2020 (CI = 2) and 08/04/2020 (CI = 3)

(D) Plot of Structural Break with segment averages for finally retained breaks

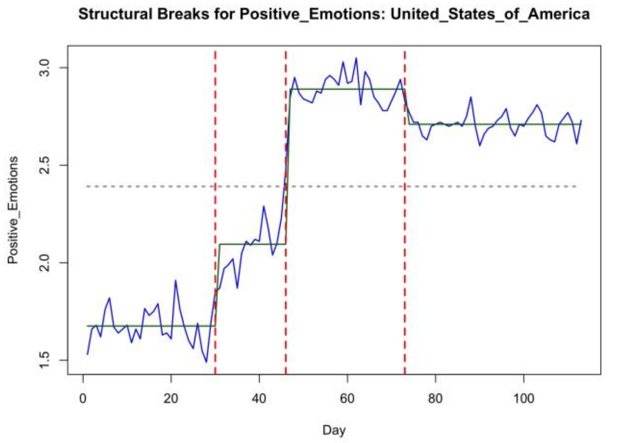

Breaks date = 25/02/2020 and 12/03/2020 and 08/04/2020

Segment averages: Segment1 = 1.68; Segment 2 = 2.10; Segment3 = 2.89; Segment 4 = 2.71

Figure 36: Plots for Anxiety - United States of America

(A) The BIC and RSS values over number of breakpoints

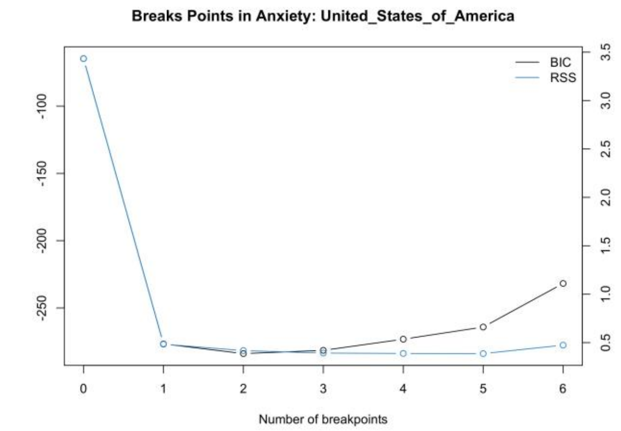

(B) Distribution of F statistics for SupF test.

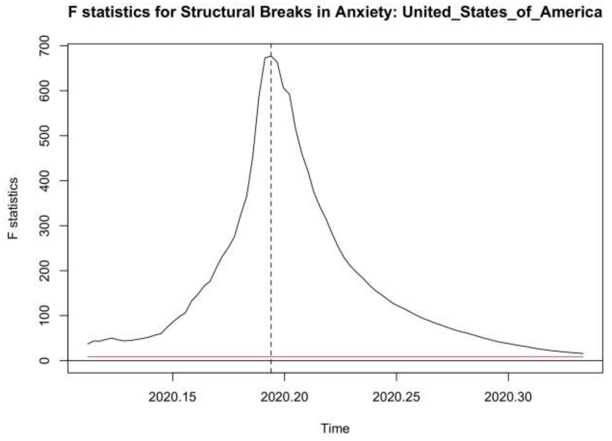

(C) BIC suggested Breaks with Confidence Interval

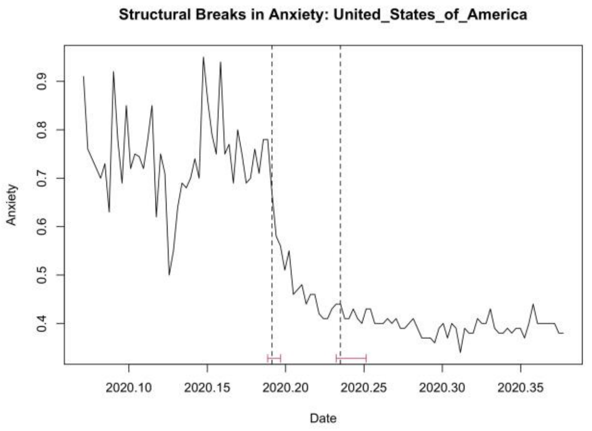

BIC suggested two breaks on 11/03/2020 (CI = 3) and 27/03/2020 (CI = 7)

(D) Plot of Structural Break with segment averages for finally retained breaks

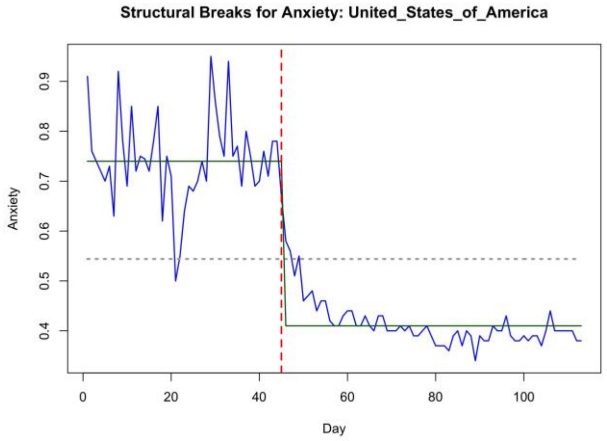

Breaks date = 11/03/2020

Segment averages: Segment1 = 0.74; Segment 2 = 0.41

Venezuela:

**Figure 37: Plots for Positive Emotion – Venezuela**	
**(A) The BIC and RSS values over number of breakpoints**<br>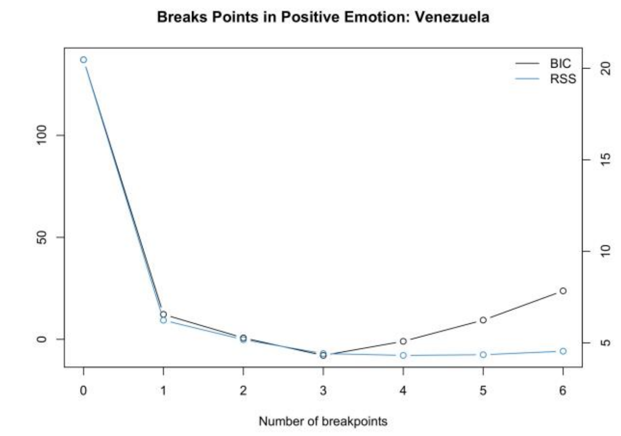	**(B) Distribution of F statistics for SupF test.**<br>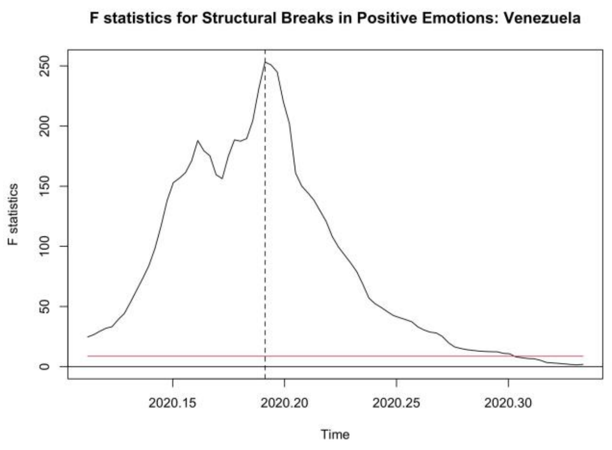
**(C) BIC suggested Breaks with Confidence Interval**<br>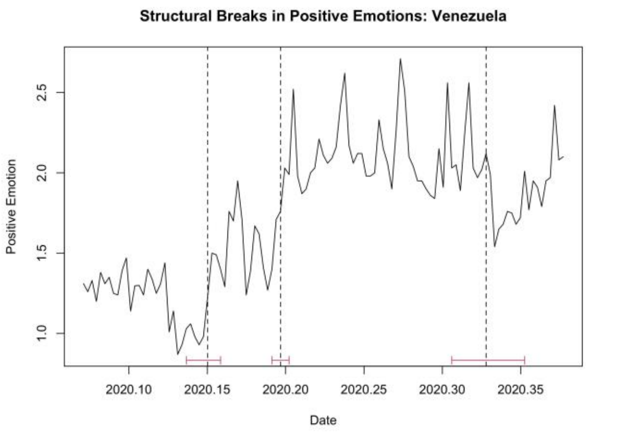<br>BIC suggested three breaks on 25/02/2020 (CI = 8) and 13/03/2020 (CI = 4) and 30/04/2020 (CI = 17)	**(D) Plot of Structural Break with segment averages for finally retained breaks**<br>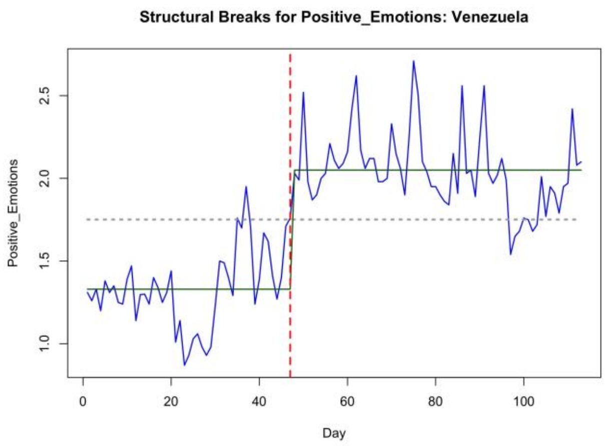<br>Breaks date = 13/03/2020<br>**Segment averages**: Segment1 = 1.33; Segment 2 = 2.05

**Figure 38: Plots for Anxiety – Venezuela**

(A) The BIC and RSS values over number of breakpoints

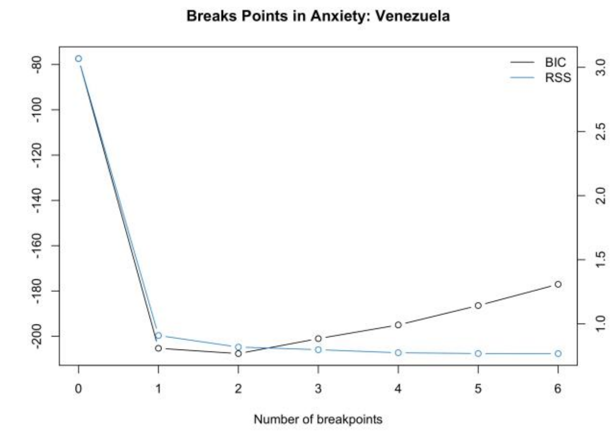

(B) Distribution of F statistics for SupF test.

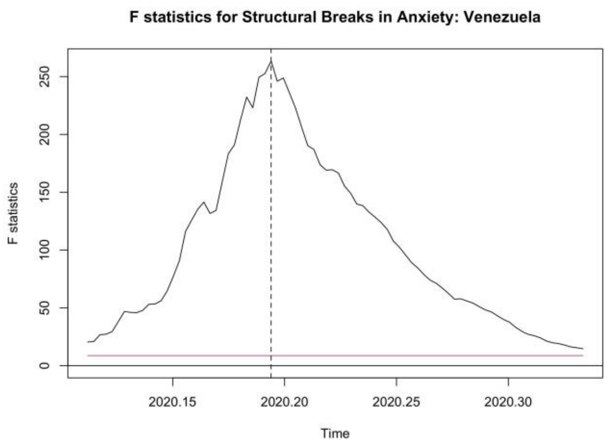

(C) BIC suggested Breaks with Confidence Interval

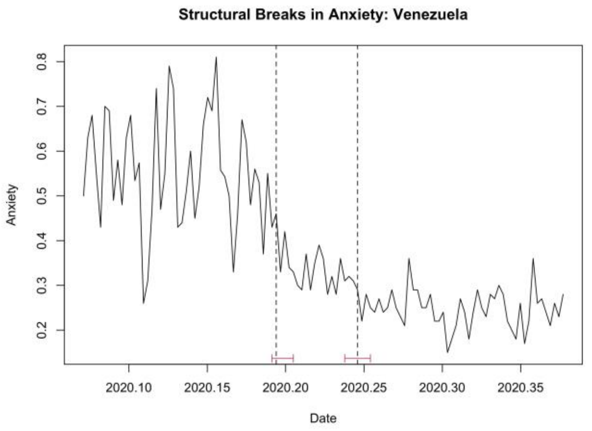

BIC suggested two breaks on 12/03/2020 (CI = 5) and 31/03/2020 (CI = 6)

(D) Plot of Structural Break with segment averages for finally retained breaks.

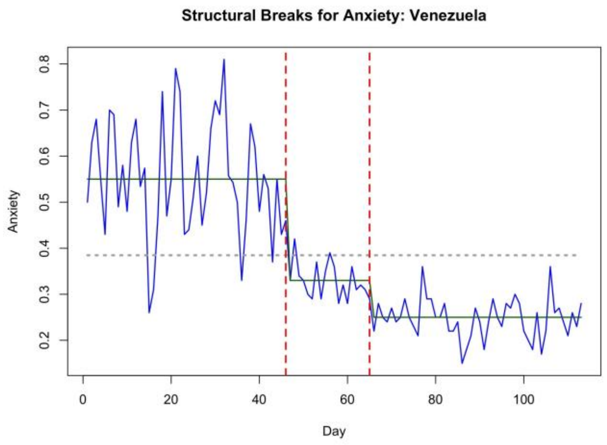

Breaks date = 12/03/2020 and 31/03/2020

Segment averages: Segment1 = 0.55; Segment 2 = 0.33; Segment 3 = .25

SUPPLEMENTARY MATERIAL 4: Structural Breaks for Anxiety and Positive Emotions (PosEmo)

Tables 4(a) to 4(f) provide detailed information about how structural breaks dates were identified. The variables (Anxiety, PosEmo) are presented separately.

Tables 4(a) and 4(b): These two tables provide *all structural break dates* suggested by Bayesian Information Criteria (BIC) for Anxiety and PosEmo, respectively. The 95% confidence interval (CI) range of dates are also presented. If the date range for 95% CI was equal to or less than 7 days, the period was retained for final discussion. The cells in the table are colour coded: dates and 95% CIs that match the retention criteria are in green whereas those that do not, are in red.

Tables 4(c) and 4(d): These two tables provide *only those structural break dates that met the retention criteria* for Anxiety and PosEmo respectively.

Tables 4(e) and 4(f): These two tables provide mean and standard deviations (SD) for the entire series, and also the segments in the series based on the break dates retained.

For example, let us look at Anxiety series for Australia. Table 4(a) shows that BIC suggested three breaks. The date-range for 95% CI of two breaks was 7 days or less but for one break (23 Feb 2020), it was 30 days. Therefore, as Table 4(c) shows, only two break dates (10th Mar 2020 & 29th Mar 2020) and their associated 95% CI date range were retained for discussion. With two structural breaks, the Anxiety series for Australia has three segments: from start of series to first SB; from first SB to second SB; and from second SB to the end of the series. Table 4(e), therefore, provides four different mean and SDs: for entire series, segment 1, segment 2, and segment 3.

Table 4a: Structural Break (SB) dates suggested by Bayesian Information Criteria (BIC) and their 95 % confidence interval for Anxiety

Country	Series Start Date	First SB Date	95% Confidence Interval	Second SB Date	95% Confidence Interval	Third SB Date	95% Confidence Interval
**Argentina**	27 Jan 2020	20 Feb 20	51 days: 19 Jan 20 to 9 Mar 20	11 Mar 20	5 days: 10 Mar 20 to 14 Mar 20		
**Australia**	27 Jan 2020	23 Feb 20	30 days: 5 Feb 20 to 5 Mar 20	10 Mar 20	6 days: 9 Mar 20 to 14 Mar 20	29 Mar 20	7 days: 28 Mar 20 to 3 Apr 20
**Canada**	27 Jan 2020	23 Feb 20	32 days: 15 Feb 20 to 17 Mar 20	11 Mar 20	3 days: 10 Mar 20 to 12 Mar 20	27 Mar 20	6 days: 26 Mar 20 to 31 Mar 20
**Colombia**	22 Feb 2020	11 Mar 20	3 days: 10 Mar 20 to 12 Mar 20	2 Apr 20	11 days: 30 Mar 20 to 9 Apr 20		
**France**	27 Jan 2020	7 Mar 20	11 days: 6 Mar 20 to 16 Mar 20	1 Apr 20	8 days: 29 Mar 20 to 5 Apr 20		
**Germany**	17 Feb 2020	10 Mar	5 days: 9 Mar 20 to 13 Mar 20				

**India**	27 Jan 2020	23 Feb 20	13 days: 15 Feb 20 t0 27 Feb 20	12 Mar 20	5 days: 11 Mar 20 to 15 Mar 20	28 Mar 20	7 days: 27 Mar 20 to 2 Apr 20
**Mexico**	27 Jan 2020	16 Mar 20	10 days: 15 Mar 20 to 24 Mar 20	1 Apr 20	6 days: 29 Mar 20 to 3 Apr 20		
**New Zealand**	2 Mar 2020	12 Mar 20	6 days: 11 Mar 20 to 16 Mar 20	24 Mar 20	5 days: 22 Mar 20 to 26 Mar 20		
**Nigeria**	27 Jan 2020	11 Mar 20	8 days: 10 Mar 20 to 17 Mar 20	27 Mar 20	6 days: 26 Mar 20 to 31 Mar 20		
**Singapore**	28 Jan 2020	6 Mar 20	14 days: 1 Mar 20 to 14 Mar 20	22 Mar 20	7 days: 21 Mar 20 to 27 Mar 20		
**South Africa**	1 Mar 2020	13 Mar 20	5 days: 12 Mar 20 to 16 Mar 20	25 Mar 20	4 days: 24 Mar 20 to 27 Mar 20		
**Spain**	23 Feb 2020	5 Mar 20	11 days: 4 Mar 20 to 14 Mar 20	17 Mar 20	5 days: 15 Mar 20 to 19 Mar 20		
**United Kingdom**	27 Jan 2020	23 Feb 20	12 days: 17 Feb 20 to 28 Feb 20	10 Mar 20	4 days: 9 Mar 20 to 12 Mar 20	26 Mar 20	5 days: 25 Mar 20 to 29 Mar 20
**United States**	27 Jan 2020	11 Mar 20	4 days: 10 Mar 20 to 13 Mar 20	27 Mar 20	8 days: 26 Mar 20 to 2 Apr 20		
**Venezuela**	27 Jan 2020	12 Mar 20	6 days: 11 Mar 20 to 16 Mar 20	31 Mar 20	7 days: 28 Mar 20 to 3 Apr 20		

Table 4b: Structural Break (SB) dates suggested by Bayesian Information Criteria (BIC) and their 95 % confidence interval for PosEmo

Country	Start Date	First SB Date	95% Confidence Interval	Second SB Date	95% Confidence Interval	Third SB Date	95% Confidence Interval
**Argentina**	27 Jan 2020	25 Feb	8 days: 23 Feb to 1 Mar	12 Mar 20	4 days: 11 Mar to 14 Mar		
**Australia**	27 Jan 2020	24 Feb 20	9 Days: 20 Feb to 28 Feb	11 Mar 20	3 days: 10 Mar 20 to 12 Mar 20		
**Canada**	27 Jan 2020	24 Feb 20	12 days: 18 Feb 20 to 29 Feb 20	12 Mar 20	3 days: 11 Mar 20 to 13 Mar 20	25 Apr 20	9 days: 19 Apr 20 to 27 Apr 20
**Colombia**	22 Feb 2020	11 Mar 20	4 days: 10 Mar 20 to 13 Mar 20				
**France**	27 Jan 2020	24 Feb 20	20 days: 17 Feb 20 to 7 Mar 20	11 Mar 20	3 days: 10 Mar 20 to 12 Mar 20	12 Apr 20	9 days: 9 Apr 20 to 17 Apr 20
**Germany**	17 Feb 2020	11 Mar 20	3 days: 10 Mar 20 to 12 Mar 20	29 Mar 20	7 days: 26 Mar 20 to 1 Apr 20		
**India**	27 Jan 2020	1 Mar 20	5 days: 27 Feb 20 to 2 Mar 20	17 Mar 20	4 days: 16 Mar 20 to 19 Mar 20	2 May 20	17 days: 23 Apr 20 to 9 May 20
**Mexico**	27 Jan 2020	24 Feb 20	19 days: 18 Feb 20 to 7 Mar 20	12 Mar 20	4 days: 11 Mar 20 to 14 Mar 20		
**New Zealand**	2 Mar 2020	12 Mar 20	4 days: 11 Mar 20 to 14 Mar 20				
**Nigeria**	27 Jan 2020	10 Mar 20	3 days: 9 Mar 20 to 11 Mar 20	5 Apr 20	21 days: 27 Mar 20 to 16 Apr 20		
**Singapore**	28 Jan 2020	10 Mar 20	3 days: 9 Mar 20 to 11 Mar 20				
**South Africa**	1 Mar 2020	12 Mar 20	3 days: 11 Mar to 13 Mar 20	6 Apr 20	21 days: 28 Mar 20 to 17 Apr 20		
**Spain**	23 Feb 2020	12 Mar 20	4 days: 11 Mar 20 to 14 Mar 20	5 Apr 20	19 days: 13 Apr 20 to 23 Apr 20		
**United Kingdom**	27 Jan 2020	25 Apr 20	8 days: 22 Feb 20 to 29 Feb 20	12 Mar 20	3 days: 11 Mar 20 to 13 Mar 20	10 Apr 20	32 days: 1 Apr 20 to 2 May 20
**United States**	27 Jan 2020	25 Feb 20	4 days: 23 Feb 20 to 26 Feb 20	12 Mar 20	3 days: 11 Mar 20 to 13 Mar 20	8 Apr 20	4 days: 7 Apr 20 to 10 Apr 20

**Venezuela**	27 Jan 2020	25 Feb 20	9 days: 20 Feb to 28 Feb	13 Mar 20	5 days: 11 Mar 20 – 15 Mar 20	30 Apr 20	18 days: 22 Apr 20 to 9 May 20

Table 4c: Structural Break dates for Anxiety retained for discussion

Country	Start Date	No. of breaks	Retained SB1		Retained SB2	
			**Break Date**	**95% Confidence Interval**	**Break Date**	**95% Confidence Interval**
**Argentina**	27 Jan 2020	*1*	11 Mar 2020	5 days: 10 Mar — 14 Mar 2020		
**Australia**	27 Jan 2020	*2*	10 Mar 2020	6 days: 9 Mar — 14 Mar 2020	29 Mar 2020	7 days: 28 Mar — 3 Apr 2020
**Canada**	27 Jan 2020	*2*	11 Mar 2020	3 days: 10 Mar — 12 Mar 2020	27 Mar 2020	6 days: 26 Mar — 31 Mar 2020
**Colombia**	22 Feb 2020	*1*	11 Mar 2020	3 days: 10 Mar — 12 Mar 2020		
**France**	27 Jan 2020	*0*				
**Germany**	17 Feb 2020	*1*	10 Mar 2020	5 days: 9 Mar —13 Mar 2020		
**India**	27 Jan 2020	*2*	12 Mar 2020	5 days: 11 Mar — 15 Mar 2020	28 Mar 2020	7 days: 27 Mar — 2 Apr 2020
**Mexico**	27 Jan 2020	*1*	01 Apr 2020	6 days: 29 Mar — 3 Apr 2020		
**New Zealand**	2 Mar 2020	*2*	12 Mar 2020	6 days: 11 Mar — 16 Mar 2020	24 Mar 2020	5 days: 22 Mar — 26 Mar 2020
**Nigeria**	27 Jan 2020	*1*	27 Mar 2020	6 days: 26 Mar — 31 Mar 2020		
**Singapore**	28 Jan 2020	*1*	22 Mar 2020	7 days: 21 Mar — 27 Mar 2020		
**South Africa**	1 Mar 2020	*2*	13 Mar 2020	5 days: 12 Mar — 16 Mar 2020	25 Mar 2020	4 days: 24 Mar — 27 Mar 2020
**Spain**	23 Feb 2020	*1*	17 Mar 2020	5 days: 15 Mar — 19 Mar 2020		
**United Kingdom**	27 Jan 2020	*1*	10 Mar 2020	4 days: 9 Mar — 12 Mar 2020		
**United States**	27 Jan 2020	*1*	11 Mar 2020	4 days: 10 Mar — 13 Mar 2020		
**Venezuela**	27 Jan 2020	*2*	12 Mar 2020	6 days: 11 Mar — 16 Mar 2020	31 Mar 2020	7 days: 28 Mar — 3 Apr 2020

Table 4d: Structural Break dates for PosEmo retained for discussion

Country	Start Date	No. of breaks	Retained SB1		Retained SB 2		Retained SB 3	
			**Break Date**	**95% Confidence Interval**	**Break Date**	**95% Confidence Interval**	**Break Date**	**95% Confidence Interval**
**Argentina**	27 Jan 2020	*1*	12 Mar 2020	4 days: 11 Mar — 14 Mar 2020				
**Australia**	27 Jan 2020	*1*	11 Mar 2020	3 days: 10 Mar — 12 Mar 2020				
**Canada**	27 Jan 2020	*1*	25 Mar 2020	6 days: 22 Mar — 27 Mar 2020				
**Colombia**	22 Feb 2020	*1*	11 Mar 2020	4 days; 10 Mar — 13 Mar 2020				
**France**	27 Jan 2020	*1*	11 Mar 2020	3 days: 10 Mar — 12 Mar 2020				
**Germany**	17 Feb 2020	*2*	11 Mar 2020	3 days: 10 Mar — 12 Mar 2020	29 Mar 2020			
**India**	27 Jan 2020	*2*	1 Mar 2020	5 days: 27 Feb — 2 Mar 2020	17 Mar 2020	4 days: 16 Mar — 19 Mar 2020		
**Mexico**	27 Jan 2020	*1*	12 Mar 2020	4 days: 11 Mar — 14 Mar 2020				

**New Zealand**	2 Mar 2020	*1*	12 Mar 2020	4 days: 11 Mar — 14 Mar 2020				
**Nigeria**	27 Jan 2020	*1*	10 Mar 2020	3 days: 9 Mar — 11 Mar 2020				
**Singapore**	28 Jan 2020	*1*	10 Mar 2020	3 days: 9 Mar — 11 Mar 2020				
**South Africa**	1 Mar 2020	*1*	12 Mar 2020	3 days: 11 Mar — 13 Mar 2020				
**Spain**	23 Feb 2020	*1*	12 Mar 2020	4 days: 11 Mar — 14 Mar 2020				
**United Kingdom**	27 Jan 2020	*1*	12 Mar 2020	3 days: 11 Mar — 13 Mar 2020				
**United States**	27 Jan 2020	*3*	25 Feb 2020	4 days: 23 Feb — 26 Feb 2020	12 Mar 2020	3 days: 11 Mar — 13 Mar 2020	8 Apr	4 days: 7 Apr — 10 Apr 2020
**Venezuela**	27 Jan 2020	*1*	13 Mar 2020	5 days: 11 Mar — 15 Mar 2020				

Table 4e: Mean and standard deviation for Anxiety time series and segments based on retained structural break dates

Country	Breaks	Entire Series		Segment One		Segment Two		Segment Three	
		Mean	SD	Mean	SD	Mean	SD	Mean	SD
**Argentina**	1	0.37	0.12	0.49	0.11	0.29	0.04		
**Australia**	2	0.59	0.20	0.78	0.16	0.46	0.08	0.43	0.03
**Canada**	2	0.56	0.18	0.76	0.11	0.43	0.06	0.40	0.03
**Colombia**	1	0.35	0.14	0.58	0.07	0.28	0.05		
**France**	0	0.37	0.10						
**Germany**	1	0.40	0.13	0.58	0.11	0.34	0.05		
**India**	2	0.51	0.24	0.75	0.20	0.36	0.10	0.31	0.03
**Mexico**	1	0.33	0.11	0.40	0.10	0.24	0.03		
**New Zealand**	2	0.48	0.17	0.83	0.17	0.49	0.08	0.39	0.05
**Nigeria**	1	0.54	0.27	0.73	0.24	0.31	0.04		
**Singapore**	1	0.52	0.18	0.66	0.15	0.38	0.06		
**South Africa**	2	0.46	0.15	0.75	0.11	0.44	0.08	0.37	0.04
**Spain**	1	0.34	0.11	0.48	0.13	0.29	0.04		
**UK**	1	0.63	0.19	0.83	0.12	0.50	0.08	0.47	0.04
**USA**	1	0.54	0.18	0.74	0.09	0.41	0.04		
Venezuela	2	0.38	0.17	0.55	0.13	0.27	0.06	0.25	0.04

Table 4f: Mean and standard deviation for PosEmo time series and segments based on retained structural break dates

Country	Breaks	Entire Series		Segment One		Segment Two		Segment Three		Segment Four	
		Mean	SD	Mean	SD	Mean	SD	Mean	SD	Mean	SD
Argentina	1	1.78	0.33	1.43	0.22	2.02	0.10				
Australia	1	2.44	0.53	1.84	0.26	2.84	0.13				
Canada	1	2.59	0.66	1.83	0.23	3.12	0.16				
Colombia	1	2.03	0.26	1.63	0.19	2.14	0.13				
France	1	2.14	0.34	1.77	0.20	2.38	0.13				
Germany	2	2.77	0.35	2.31	0.22	3.01	0.23				
India	2	2.48	0.63	1.67	0.19	2.81	0.41	2.99	0.16		
Mexico	1	1.84	0.38	1.43	0.23	2.12	0.12				
New Zealand	1	2.73	0.24	2.27	0.21	2.81	0.15				
Nigeria	1	2.36	0.62	1.69	0.29	2.79	0.32				
Singapore	1	2.42	0.40	1.97	0.20	2.70	0.18				
South Africa	1	3.08	0.47	2.10	0.22	3.26	0.22				
Spain	1	2.03	0.23	1.66	0.20	2.13	0.10				
UK	1	2.73	0.72	1.91	0.26	3.30	0.19				
USA	3	2.39	0.50	1.68	0.10	2.60	0.39	2.78	0.11	2.71	0.05
Venezuela	1	1.75	0.43	1.33	0.24	2.05	0.24				
